# Cavity-Containing [Fe_2_L_3_]^4+^ Helicates: An Examination of Host-Guest Chemistry and Cytotoxicity

**DOI:** 10.3389/fchem.2021.697684

**Published:** 2021-07-07

**Authors:** Lynn S. Lisboa, Mie Riisom, Roan A. S. Vasdev, Stephen M. F. Jamieson, L. James Wright, Christian G. Hartinger, James D. Crowley

**Affiliations:** ^1^ Department of Chemistry, University of Otago, Dunedin, New Zealand; ^2^ School of Chemical Sciences, University of Auckland, Auckland, New Zealand; ^3^ Auckland Cancer Society Research Centre, University of Auckland, Auckland, New Zealand

**Keywords:** iron(II), helicate, cytotoxicity, host-guest chemistry, metallosupramolecular architectures

## Abstract

Two new di(2,2′-bipyridine) ligands, 2,6-bis([2,2′-bipyridin]-5-ylethynyl)pyridine (**L1**) and bis(4-([2,2′-bipyridin]-5-ylethynyl)phenyl)methane (**L2**) were synthesized and used to generate two metallosupramolecular [Fe_2_(**L**)_3_](BF_4_)_4_ cylinders. The ligands and cylinders were characterized using elemental analysis, electrospray ionization mass spectrometry, UV-vis, ^1^H-, ^13^C and DOSY nuclear magnetic resonance (NMR) spectroscopies. The molecular structures of the [Fe_2_(**L**)_3_](BF_4_)_4_ cylinders were confirmed using X-ray crystallography. Both the [Fe_2_(**L1**)_3_](BF_4_)_4_ and [Fe_2_(**L2**)_3_](BF_4_)_4_ complexes crystallized as racemic (*rac*) mixtures of the ΔΔ (P) and ΛΛ (M) helicates. However, ^1^H NMR spectra showed that in solution the larger [Fe_2_(**L2**)_3_](BF_4_)_4_ was a mixture of the *rac*-ΔΔ/ΛΛ and *meso*-ΔΛ isomers. The host-guest chemistry of the helicates, which both feature a central cavity, was examined with several small drug molecules. However, none of the potential guests were found to bind within the helicates. *In vitro* cytotoxicity assays demonstrated that both helicates were active against four cancer cell lines. The smaller [Fe_2_(**L1**)_3_](BF_4_)_4_ system displayed low μM activity against the HCT116 (IC_50_ = 7.1 ± 0.5 μM) and NCI-H460 (IC_50_ = 4.9 ± 0.4 μM) cancer cells. While the antiproliferative effects against all the cell lines examined were less than the well-known anticancer drug cisplatin, their modes of action would be expected to be very different.

## Introduction

**FIGURE 1:**
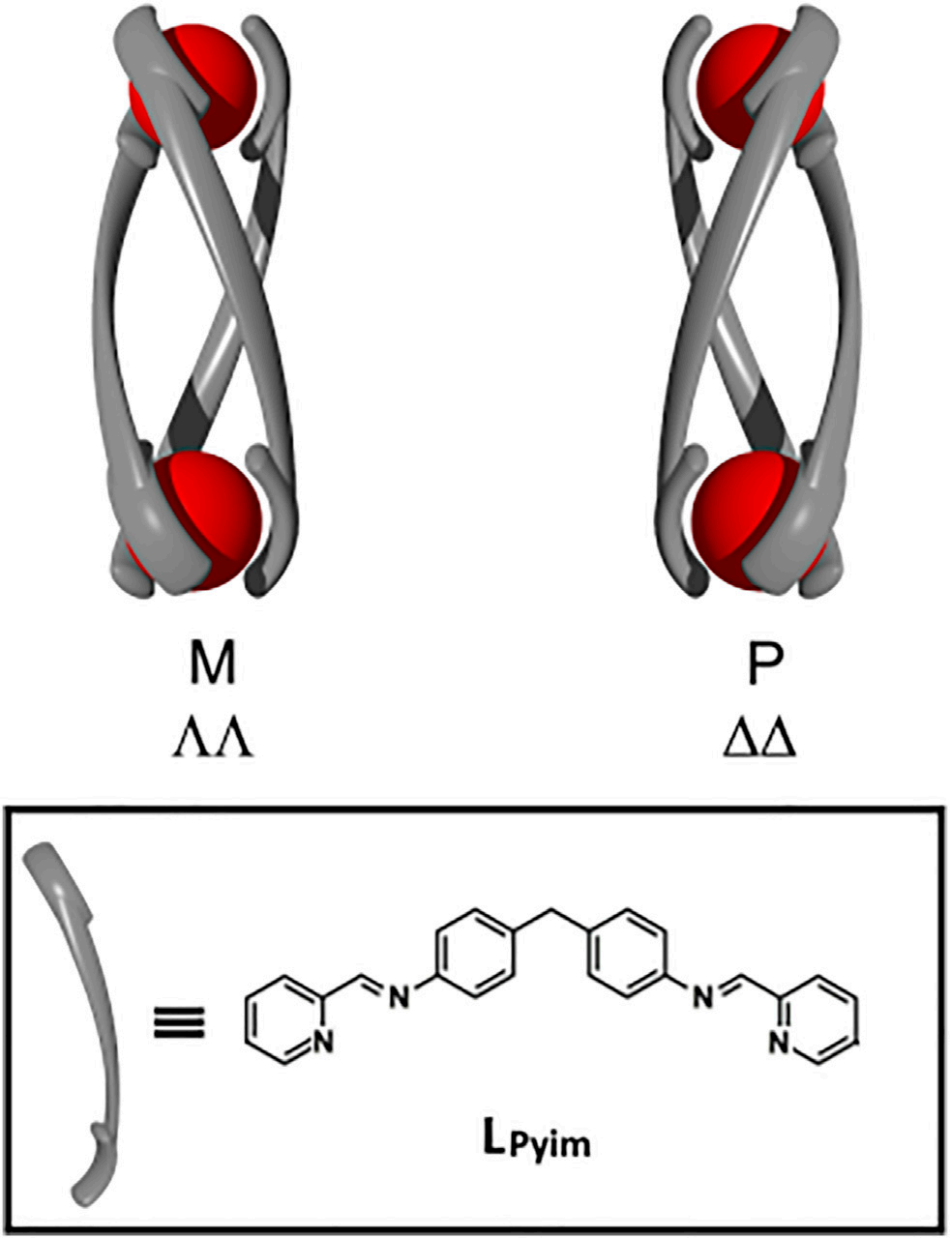
Cartoon representations of the minus (M, ΛΛ), plus (P, ΔΔ) helicate isomers of a generic triple-stranded helicate and the chemical structure of the (1E,1′E)-N,N′-[methylenebis(4,1-phenylene)]bis[1-(pyridin-2-yl)methanimine] ligand (**L**_**Pyim**_) developed by Hannon and co-workers (Hannon et al., 1997).

Metallosupramolecular architectures (MSAs) are beginning to display a wide range of applications (Yoshizawa et al., 2009; Cook and Stang, 2015; Hong et al., 2018; Saha et al., 2018; Bardhan and Chand, 2019; Gao et al., 2019; Rizzuto et al., 2019; Percastegui et al., 2020) Largely inspired by the success of small molecule metallo-drugs (Hartinger and Dyson, 2009; Mjos and Orvig, 2014; Anthony et al., 2020; Boros et al., 2020; Frei, 2020; Frei et al., 2020; Steel et al., 2021b) there is a growing interest in biological applications of MSAs (Cook et al., 2013; Therrien, 2015; Pöthig and Casini, 2019; Sepehrpour et al., 2019; Samanta and Isaacs, 2020). Systems have been studied for their anti-cancer and anti-microbial activity and their potential as drug delivery agents. Helicates (Piguet et al., 1997; Albrecht, 2001; Hannon and Childs, 2004; Howson and Scott, 2011; Boiocchi and Fabbrizzi, 2014; Paneerselvam et al., 2018; Albrecht et al., 2019; Albrecht, 2020; Tran and Yoo, 2020) are one of the earlier known and well-studied sub-classes of MSAs. Lehn and co-workers reported the first helicates; a double-stranded dinuclear and larger trinuclear system were generated from poly(2,2′-bipyridine) ligands and Cu(I) ions (Lehn et al., 1987) Subsequently, single, triple, and quadruple stranded helicates have all been synthesized and these systems are chiral featuring P (plus, right-handed) and M (minus, left-handed) helices (Figure 1). Because of the structural relationship to helical natural materials such as DNA, α-helices and zinc fingers of proteins there has been considerable interest in the biological properties, of helicates. Early work by Lehn and co-workers showed that double-stranded helicates assembled from poly(2,2′-bipyridine) ligands and Cu(I) ions could bind to double stranded DNA (Schoentjes and Lehn, 1995). Others have examined DNA binding and nuclease activity (Childs et al., 2006), and the cytotoxicity of related double-stranded complexes (Holtze et al., 2006; Allison et al., 2018). In addition, quadruple-stranded helicates have been shown to be cytotoxic (Mcneill et al., 2015; Ahmedova et al., 2016a; Ahmedova et al., 2016b; Schmidt et al., 2016; Vasdev et al., 2018; Ahmedova et al., 2020) and in some cases the modes of action of the complexes have been studied (Mcneill et al., 2020).

While there are only a few reports on the biological properties of double- and quadruple-stranded helicates, the related triple-stranded analogues have been extensively examined. These triply-stranded supramolecular structures are assembled from an octahedral metal ion and di(bidentate) linker ligands [M_2_L_3_]; this combination of building blocks can generate three isomeric complexes the chiral M (ΛΛ) and P (ΔΔ), and the *meso* (ΛΔ). Pioneering work by Hannon and co-workers described the synthesis of the first [Fe_2_(**L**_**Pyim**_)_3_]^4+^ helicates (where **L**_**Pyim**_ = (1E,1′E)-N,N′-[methylenebis(4,1-phenylene)]bis[1-(pyridin-2-yl)methanimine, Figure 1] obtained from pyridylimine binding motifs, octahedral Fe(II) ions and a diphenylmethylene spacer unit (Hannon et al., 1997). The mechanical coupling exerted by the spacer unit meant that a racemic (*rac*) mixture of the M (ΛΛ) and P (ΔΔ) [M_2_(**L**_**Pyim**_)_3_]^4+^ helicates (where M = Fe(II) or Ni(II)) was formed and the authors went on to show that the M and P helicates could be resolved by chiral chromatography (Hannon et al., 2001a). The interaction of the M− and P−[Fe_2_(**L**_**Pyim**_)_3_]^4+^ helicates with DNA has been extensively examined. The complexes have been shown to bind in the major grove of duplex DNA (Moldrheim et al., 2002), and at the center of three-way DNA (Oleksi et al., 2006; Cerasino et al., 2007; Malina et al., 2007; Cardo et al., 2011) and RNA (Phongtongpasuk et al., 2013) junctions (3WJ). More recently, the [Fe_2_(**L**_**Pyim**_)_3_]^4+^ helicates were also shown to bind to DNA and RNA bulges (Malina et al., 2014; Malina et al., 2016). Similar observations have been made with the related [M_2_(**L**_**Pyim**_)_3_]^4+^ helicates [where M = Ru(II) or Ni(II)] (Cardo et al., 2018) and the Ni(II) and Fe(II) helicates have been demonstrated to interact with G-quadruplexes (Zhao et al., 2013; Zhao et al., 2016) and the β-amyloid polypeptide (Aβ) (Yu et al., 2012; Li et al., 2015). Furthermore, the interaction of the iron(II) helicate with duplex DNA induces intramolecular DNA coiling (Hannon et al., 2001b; Malina et al., 2008) and it has been shown to display anti-cancer (Hotze et al., 2008), anti-bacterial (Richards et al., 2009) and anti-fungal (Vellas et al., 2013) properties, but is not mutagenic or genotoxic. This remarkable range of biological properties has been obtained without investigating changes to either the metal binding (pyridylimine) or spacer units of the helicates, suggesting that the system could potentially be improved by further tuning of the molecular scaffold.

Building on the aforenoted work, Scott and co-workers developed an excellent method for the self-assembly of optically pure single diastereomer *fac*-[Fe(**L**_**pyimR**_)_3_]^2+^ (where **L**_**pyimR**_ = functionalized pyridylimine ligand) complexes (Howson et al., 2009). The same group then exploited this method to synthesize enantiomerically pure Fe(II) and Zn(II) [M_2_(**L**_**pyimR**_)_3_]^4+^ helicates and flexicates which feature different spacer systems. Like the parent Hannon helicate, it has been shown that these new pyridylimine-based complexes have shown a diverse range of biological properties and some flexicates have impressive, tunable anti-microbial (Howson et al., 2012; Simpson et al., 2019) and anti-cancer (Brabec et al., 2013; Faulkner et al., 2014; Kaner and Scott, 2015; Kaner et al., 2016; Song et al., 2019; Song et al., 2020) properties. The interactions of this more diverse family of helicates with DNA/RNA (Song et al., 2021) and proteins (Li et al., 2014) have also been examined and the systems show structure-dependent binding to duplex DNA (Brabec et al., 2013; Malina et al., 2015b), G-quadruplexes (Zhao et al., 2017; Zhao et al., 2018), 3WJ and 4WJ (Brabec et al., 2013), and bulges (Malina et al., 2015a; Hrabina et al., 2020).

The success of the pyridylimine [M_2_L_3_]^4+^ helicates discussed above has inspired others to examine the biological properties of related [M_2_L_3_]^4+^ triply-stranded helicates. For example, we have explored the use of small families of di(2-pyridyl-1,2,3-triazole) ligands (**L**_**dipytri**_) to generate Fe(II), Ru(II), and Co(III) helicates (Vellas et al., 2013; Kumar et al., 2015; Vasdev et al., 2016). The biological properties of the Fe(II) and Ru(II) systems were poor but the more robust Co(III) helicates were shown to bind to and condense DNA and in addition displayed good anticancer activity (Crlikova et al., 2020). Di(2,2′-bipyridine) ligands (Glasson et al., 2008; Glasson et al., 2011a; Glasson et al., 2011b) have also been used to generate [M_2_L_3_]^4+^ triple-stranded helicates and recently Vázquez and co-workers have examined the DNA binding and cytotoxicity of some peptide linked [M_2_L_3_]^4+^ helicates [where M = Fe(II) or Co(III)] (Gamba et al., 2014; Gómez-González et al., 2018; Gomez-Gonzalez et al., 2021).

Given the well demonstrated ability of [M_2_L_3_]^4+^ helicates to bind to DNA/RNA and proteins and their potential to be used as targeted therapeutics, we herein report the synthesis of two new di(2,2′-bipyridine) ligands, 2,6-bis([2,2′-bipyridin]-5-ylethynyl)-pyridine (**L1**) and bis(4-([2,2′-bipyridin]-5-ylethynyl)phenyl)-methane (**L2**) and their use in the assembly of two new triple-stranded [Fe_2_L_3_]X_4_ helicates (X = BF_4_^−^, OTf^−^ or Cl^−^). Moreover, due the presence of a central cavity in both the [Fe_2_(**L1**)_3_](BF_4_)_4_ and [Fe_2_(**L2**)_3_](BF_4_)_4_ helicates, we also report our examination of the host-guest properties of these systems with some small molecule drugs and our studies of the anti-cancer activity of the complexes.

## Results and Discussion

The new di(2,2′-bipyridine) ligands, 2,6-bis([2,2′-bipyridin]-5-ylethynyl)-pyridine (**L1**) and bis(4-([2,2′-bipyridin]-5-ylethynyl)-phenyl)methane (**L2**) were synthesized from 5-ethynyl-2,2′-bipyridine (Grosshenny et al., 1997) and either 2,5-dibromopyridine or bis(4-iodophenyl)methane (Austin et al., 1981) using standard Sonogashira cross-coupling conditions (Supplementary Material) and were obtained in modest yields (**L1** = 51% and **L2** = 61%). The ligands were characterized using ^1^H nuclear magnetic resonance (NMR), ^13^C{^1^H} NMR, electrospray ionization mass spectrometry (ESIMS) and elemental analysis (Supplementary Figures S1–S4).

**SCHEME 1:**
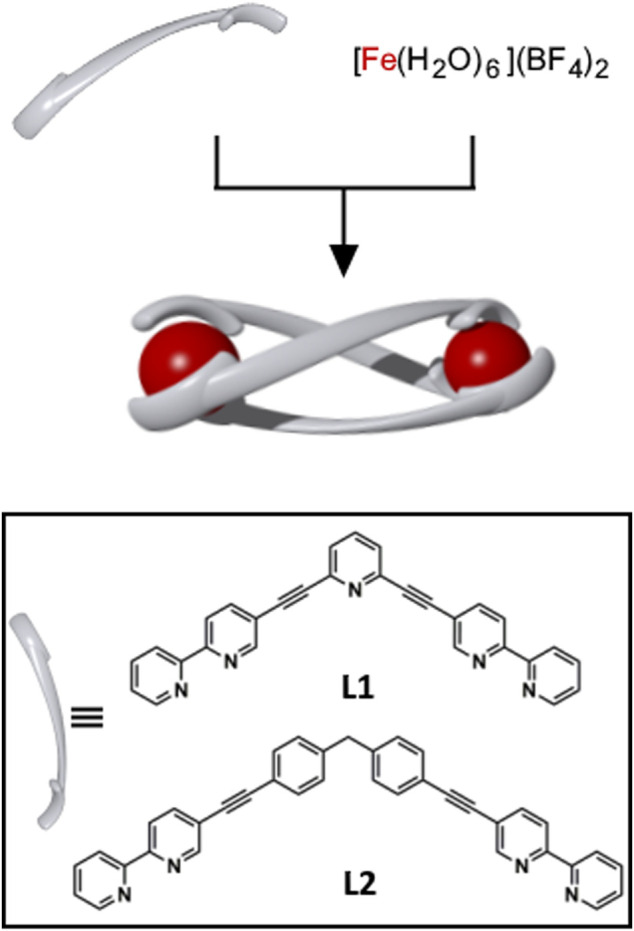
Cartoon representation of the synthesis of the triply-stranded metallo-cylinders [Fe_2_(**L1**)_3_](BF_4_)_4_ and [Fe_2_(**L2**)_3_](BF_4_)_4_. The complexes were synthesized by combining **L1** or **L2** (3 equiv.) with [Fe(H_2_O)_6_](BF_4_)_2_ (2 equiv.), CH_3_CN, 65°C, 16 h. Bottom: inset showing the structures of **L1** and **L2**.

The [Fe_2_(**L**)_3_](BF_4_)_4_ helicates were synthesised by combining either **L1** or **L2** (3 equiv.) with [Fe(H_2_O)_6_](BF_4_)_2_ (2 equiv.) in acetonitrile at 65°C (Scheme 1 and Supplementary Material). The ligands were initially insoluble in the reaction mixture, however, after 5 min the ligands dissolved and deep red solutions (*λ*_max_ = 545 or 547 nm, respectively) were obtained. The resulting Fe(II) complexes were purified by recrystallization (vapour diffusion of diethyl ether into a nitromethane solution) and deep red crystals were isolated in good yields (89% for [Fe_2_(**L1**)_3_](BF_4_)_4_ and 77% for [Fe_2_(**L2**)_3_](BF_4_)_4_). The complexes were characterised by ^1^H NMR, ^13^C{^1^H} NMR, ^1^H DOSY NMR, UV-vis spectroscopy, ESIMS, and X-ray crystallography (Supplementary Material).

The ESIMS data obtained for the two complexes displayed a major peak consistent with the corresponding [Fe_2_ **L**_3_]^4+^ cation (m/z = 354.5886 [Fe_2_(**L1**)_3_]^4+^ and m/z = 421.3972 m/z [Fe_2_(**L2**)_3_]^4+^, respectively) suggestive of the formation of the expected triple-stranded helicates (Figure, Supplementary Figures S7, S10). The ^1^H DOSY NMR spectra (500 MHz, CD_3_CN, 298 K) of [Fe_2_(**L1**)_3_](BF_4_)_4_ and [Fe_2_(**L2**)_3_](BF_4_)_4_ were collected, and all the proton resonances displayed the same diffusion coefficients (6.51 ± 0.03 × 10^−10^ m^2^ s^−1^ and 5.40 ± 0.10 × 10^−10^ m^2^ s^−1^, respectively) suggesting that a single metallosupramolecular architecture or mixtures of isomeric architectures were obtained (Supplementary Figures S11, S12). In addition, the observed diffusion coefficients were similar to those found for some related [Fe_2_(**L**_**dipytri**_)_3_]^4+^ metallo-cylinders (Vellas et al., 2013) providing further support for the formation of the desired triple-stranded helicates.

**FIGURE 2:**
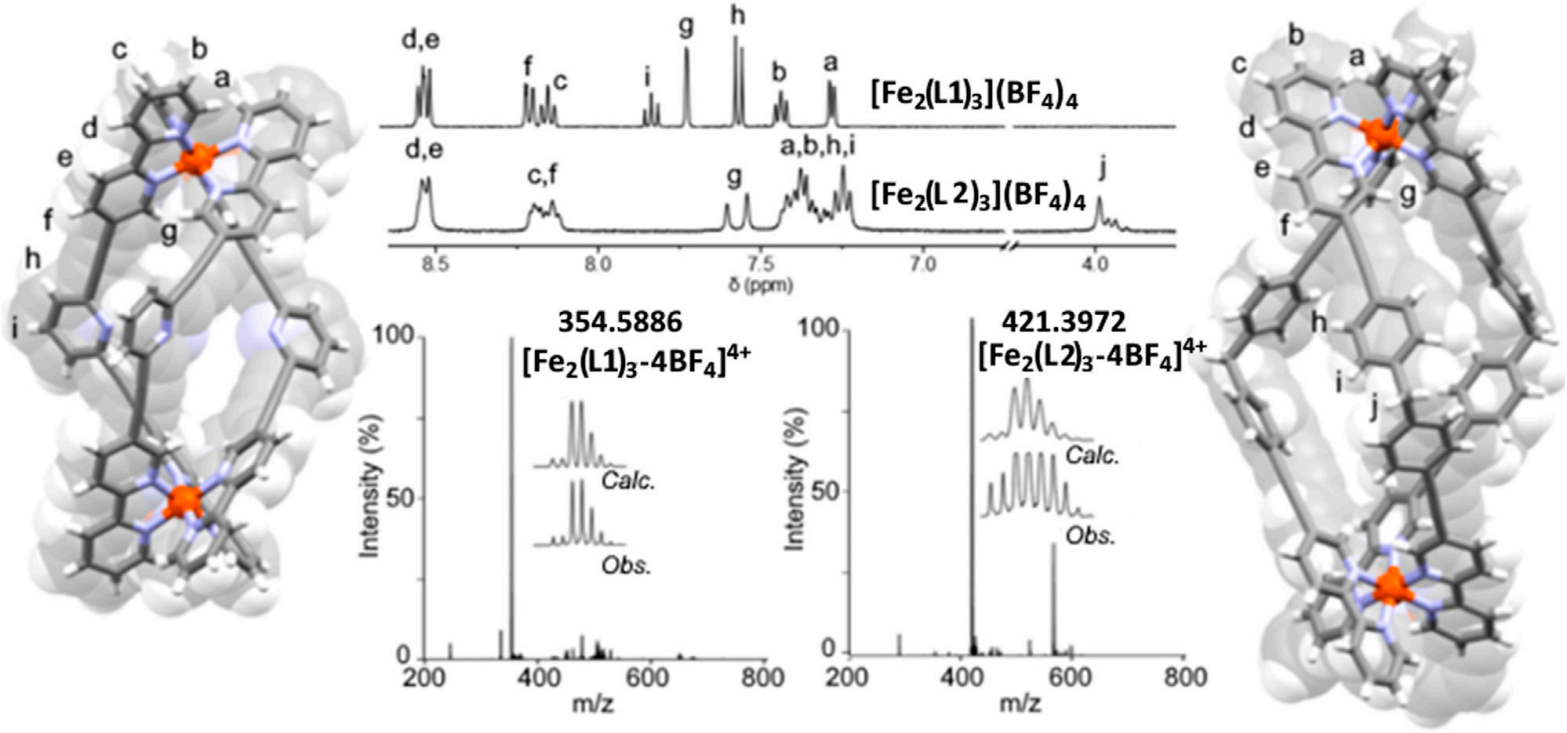
X-ray structures showing the overlaid stick and spacefill representations of [Fe_2_(**L1**)_3_](BF_4_)_4_ and [Fe_2_(**L2**)_3_](BF_4_)_4_ with related partial ^1^H NMR spectra (500 MHz, CD_3_CN, 298 K) and associated ESI-mass spectra. Fe-Fe distances; [Fe_2_(**L1**)_3_](BF_4_)_4_ = 14.2 Å, [Fe_2_(**L2**)_3_](BF_4_)_4_ = 19.1 Å. Solvent molecules and counter anions were omitted for clarity.

The ^1^H NMR spectra (500 MHz, CD_3_CN, 298 K) for the complexes of **L1** and **L2** were significantly distinct (Figure 2; Supplementary Material). The spectrum of [Fe_2_(**L1**)_3_](BF_4_)_4_ displayed nine sharp resonances in the aromatic region (*δ* = 8.5−7.0 ppm) consistent with the formation of a racemic (*rac*) mixture of the helical [Fe_2_(**L1**)_3_]^4+^ isomers, P = ΔΔ and M = ΛΛ. Conversely, the ^1^H NMR spectrum of [Fe_2_(**L2**)_3_](BF_4_)_4_ was more complex with several broad overlapping resonances in the aromatic region. However, the methylene protons of the spacer backbone (H_j,_ *δ* = 4.0−3.8 ppm) were clearly split into two distinct resonances; a singlet and an AB quartet. This suggests that in solution [Fe_2_(**L2**)_3_](BF_4_)_4_ forms a mixture of helicate *rac-*ΔΔ/ΛΛ and mesocate *meso-*ΔΛ isomers. Others (Goetz and Kruger, 2006; Vellas et al., 2013) have observed this behavior in solution with related [Fe_2_ **L**_3_]^4+^ systems that feature the diphenylmethylene spacer unit. This is in contrast to observations with the pyridyl imine helicate, [Fe_2_(**L_Pyim_**)_3_]^4+^ of Hannon and co-workers (Hannon et al., 1997). Those helicates have the same spacer unit and are found to exclusively form *rac*-helicates in solution and the solid state. The difference appears to be related to the larger size of [Fe_2_(**L2**)_3_]^4+^ compared to [Fe_2_(**L**_**Pyim**_)_3_]^4+^. In [Fe_2_(**L**_**Pyim**_)_3_]^4+^, the aryl rings of the spacer unit are in close contact and interdigitate, mechanically locking the complex into the helical arrangement. The larger size of **L2** lessens this steric interdigitation of the spacer aryl groups, therefore making the mesocate arrangement more energetically accessible.

The molecular structures of [Fe_2_(**L1**)_3_](BF_4_)_4_ and [Fe_2_(**L2**)_3_](BF_4_)_4_ were confirmed by X-ray crystallography with crystals grown by slow vapour diffusion of diethyl ether into nitromethane solutions (Figure 2, Supplementary Figures S27, S28). The [Fe_2_(**L1**)_3_](BF_4_)_4_ structure was solved in the *P* $\bar{1}\;$space group and the asymmetric unit contains two iron ions, three **L1** ligands, four tetrafluoroborate anions and two nitromethane solvent molecules. Each iron(II) ion is coordinated to three bipy units generating a triple-stranded helicate architecture. The Fe-Fe distance of 14.2 Å confirmed that the system is elongated in comparison to the parent Hannon helicate. The compound crystallized as a racemic mixture in the solid state with both the ΔΔ (P) and ΛΛ (M) isomers (see the structural representations in Figure 2) present in the crystal.

The [Fe_2_(**L2**)_3_](BF_4_)_4_ structure was solved in the *P*2_1_/*n* space group and the asymmetric unit was occupied by one [Fe_2_(**L2**)_3_]^4+^ unit, two tetrafluoroborate anions and seven co-crystallized nitromethane molecules. [Fe_2_(**L2**)_3_](BF_4_)_4_ was shown to crystallize as a *rac* mixture of the ΔΔ (P) and ΛΛ (M) helicates. The *meso*-form detected in solution by ^1^H NMR spectroscopy was not observed in the solid state, presumably due to crystal packing effects (Vellas et al., 2013). The combination of the two 5-ethynyl-2,2′-bipyridine units and the diphenylmethylene spacer in **L2** led to a large Fe-Fe distance (19.1 Å) in [Fe_2_(**L2**)_3_](BF_4_)_4._ The metallo-architectures of both [Fe_2_(**L1**)_3_](BF_4_)_4_ and [Fe_2_(**L2**)_3_](BF_4_)_4_ displayed a clear central cavity and notable π surfaces available for potential host-guest interactions (Figure 2; Supplementary Material).

**FIGURE 3:**
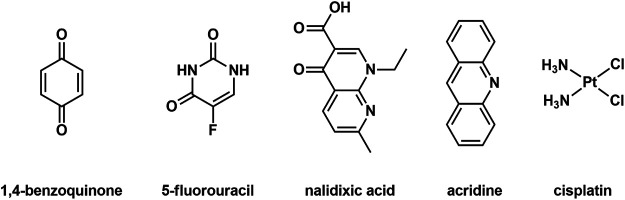
Guest molecules examined in the host-guest study with the helicates.

MSA cage systems have been used extensively as hosts for small molecule guests (Yoshizawa et al., 2009; Cook and Stang, 2015; Hong et al., 2018; Saha et al., 2018; Bardhan and Chand, 2019; Gao et al., 2019; Rizzuto et al., 2019; Percastegui et al., 2020). In contrast, the use of [Fe_2_ **L**_3_]^4+^ helicate architectures as hosts is far less common, presumably because the vast majority of reported MSAs do not contain a central cavity. Recently, there have been a few reports of guest binding [anions (Goetz and Kruger, 2006; Cui et al., 2012), sugars (Yang et al., 2021) and small aromatic molecules (Fazio et al., 2018; Jiang et al., 2019)] within [Fe_2_ **L**_3_]^4+^ helicates that feature small cavities. As the cavities of the [Fe_2_(**L1**)_3_]^4+^ and [Fe_2_(**L2**)_3_]^4+^ helicates are both lined by functional groups that could interact with guests through either hydrogen bonding or π-interactions, we sought out some small drug molecules that could potentially interact with the helicates using those non-covalent interactions. Therefore, the guest molecules 1,4-benzoquinone, nalidixic acid, acridine (as an analogue of proflavine), cisplatin and 5-fluorouracil were selected as they are either known or analogues of known anticancer and antibacterial drugs (Figure 3; Supplementary Material). The host-guest (HG) interactions were examined using ^1^H NMR spectroscopy and ESIMS. One of the potential guest molecules (2 equiv.) was combined with one of the helicates (1 equiv.) in CD_3_CN at 298 K and the ^1^H NMR spectrum acquired (Supplementary Material). ^1^H NMR spectra of the host-guest mixtures were then compared to the ^1^H NMR spectra of the corresponding “free” host and guest compounds (Supplementary Figures S23, S24). Disappointingly, no complexation induced shifts were observed for either the host or the guest resonances suggesting that none of the guests bound within the cavities of the helicates. Molecular models (SPARTAN16, MMFF, Supplementary Figures S25, S26) showed that there are no obvious steric interactions that would prevent host-guest formation for the majority of the examined HG pairs. Thus, the lack of guest binding in the cases examined appears to be due to the absence of the correct combination of complementary non-covalent and solvaphobic interactions. Additionally, the BF_4_^−^ counter-anions may be competing for the cavity as has been observed in other cationic MSA systems (August et al., 2016).

Related [Fe_2_ **L**_3_]^4+^ helicates have shown excellent anticancer activity (Song et al., 2021). Therefore, we examined the cytotoxicity of [Fe_2_(**L1**)_3_]^4+^ and [Fe_2_(**L2**)_3_]^4+^ against a panel of cancer cell lines. As the [Fe_2_(**L1**)_3_](BF_4_)_4_ and [Fe_2_(**L2**)_3_](BF_4_)_4_ complexes were only soluble in polar organic solvents (DMSO, CH_3_CN, CH_3_NO_2,_ and acetone) we attempted to render the helicates water soluble by exchanging the BF_4_^−^ counter anions with Cl^−^ or OTf^−^ (Supplementary Material). While we were able to generate the new [Fe_2_(**L1**)_3_](X)_4_ and [Fe_2_(**L2**)_3_](X)_4_ (where X = Cl^−^, OTf^−^) salts, they proved even less soluble than the original BF_4_^−^ salts. The OTf^−^ salts were soluble in CH_3_CN and DMSO, however, the Cl^−^ salts were only soluble in DMSO with none of the systems showing any appreciable water solubility (Supplementary Figures S18–S21). Due to these complications, we carried out the cytotoxicity experiments with [Fe_2_(**L1**)_3_](BF_4_)_4_ and [Fe_2_(**L2**)_3_](BF_4_)_4_ dissolved in DMSO and these solutions were then diluted with biological media to the required concentrations.

Due to the modest water solubility of many drug candidates, it is common to use DMSO to solubilize compounds for cytotoxicity experiments. However, it is also well known that DMSO can displace coordinated ligands and decompose metal complexes (Patra et al., 2013; Vellas et al., 2013; Hall et al., 2014; Huang et al., 2017). Therefore, the stability of the helicates in neat DMSO and a 1:19 v/v DMSO:water mixture was examined before carrying out the cytotoxicity experiments. The stabilities of [Fe_2_(**L1**)_3_](BF_4_)_4_ and [Fe_2_(**L2**)_3_](BF_4_)_4_ in these solvents were monitored using ^1^H NMR and UV-vis spectroscopy (Supplementary Figures S14, S15). Both complexes completely decomposed (*ca*. 3 h for [Fe_2_(**L1**)_3_](BF_4_)_4_ and 24 h for [Fe_2_(**L2**)_3_](BF_4_)_4_) in neat DMSO liberating the free ligands and presumably forming [Fe(DMSO)_6_]^2+^ (White et al., 2007).

The helicates were more long lived in 1:19 v/v DMSO:water mixtures. A 72 h UV-visible stability study of [Fe_2_(**L1**)_3_](BF_4_)_4_ and [Fe_2_(**L2**)_3_](BF_4_)_4_ in 1:19 v/v DMSO:water was conducted to replicate the timeframe of the biological testing (Supplementary Figures S16, S17). Interestingly, the smaller [Fe_2_(**L1**)_3_](BF_4_)_4_ showed no signs of decomposition (within the uncertainty of the measurement) whereas the larger [Fe_2_(**L2**)_3_](BF_4_)_4_ did slowly degrade, approximately 43% of the [Fe_2_(**L2**)_3_](BF_4_)_4_ was still present in solution after 72 h. Given the moderate to good stability of the helicates under conditions similar to those required for the cytotoxicity assay we proceeded to measure the *in vitro* antiproliferative activity of the compounds.

The ligands (**L1** and **L2**) and helicates [Fe_2_(**L1**)_3_](BF_4_)_4_ and [Fe_2_(**L2**)_3_](BF_4_)_4_ were subjected to the sulforhodamine B cytotoxicity assay in the human cancer cell lines HCT116 (colorectal carcinoma), NCI-H460 (non-small cell lung carcinoma), SiHa (cervical carcinoma), and SW480 (colon adenocarcinoma) (Table 1). **L1** and **L2** proved to be insoluble under the conditions of the experiments, and therefore their antiproliferative activity could not be determined (Table 1). The helicates [Fe_2_(**L1**)_3_](BF_4_)_4_ and [Fe_2_(**L2**)_3_](BF_4_)_4_ were both active *in vitro* against all the cancer cells tested. The smaller [Fe_2_(**L1**)_3_](BF_4_)_4_ was more active than [Fe_2_(**L2**)_3_](BF_4_)_4_ against all the cell lines examined, and displayed low μM activity against HCT116 (IC_50_ = 7.1 ± 0.5 μM) and NCI-H460 (IC_50_ = 4.9 ± 0.4 μM) cancer cells. Unfortunately, direct comparisons with the previously studied isostructural helicates [Fe_2_(**L**_**Pyim**_)_3_]^4+^ and [Fe(**L**_**pyimR**_)_3_]^2+^ are difficult as their cytotoxicity was determined with different cell lines. However, the low μM activity observed for [Fe_2_(**L1**)_3_](BF_4_)_4_ suggests that it could be more active than the [Fe_2_(**L**_**Pyim**_)_3_]^4+^ helicates (IC_50_ values ranged from 19–52 μM, despite having been determined in different cell lines) (Hotze et al., 2008). The observed activity of [Fe_2_(**L1**)_3_](BF_4_)_4_ is similar in magnitude to that found by Scott et al. for their family of [Fe_2_(**L**_**pyimR**_)_3_]^2+^ helicates (Song et al., 2021). However, the [Fe_2_(**L**_**pyimR**_)_3_]^2+^ systems are more effective overall with some of that family displaying nanomolar activities (Kaner et al., 2016; Song et al., 2020). We have recently studied the cytotoxicity of a small family of dimetallic organometallic (Ru, Rh, Os, and Ir) complexes (Steel et al., 2021a) of **L**_**Pyim**_ against the same series of cell lines enabling a more direct comparison (Table 1). Both helicates displayed better activity than the dimetallic complexes and **L**_**Pyim**_ across the range of cell lines. Presumably the higher activity of the helicates is associated with the different molecular shape and higher charge. While the *in vitro* activity of the [Fe_2_(**L1**)_3_](BF_4_)_4_ helicate is promising we note that the widely used anti-cancer drug cisplatin is more active in all the cell lines examined (Table 1). However, the mode of action of this covalent DNA binder, in comparison to supramolecular structures that are more likely to form non-covalent interactions with biological targets, will be very different, making any direct comparison difficult.

**TABLE 1:** IC_50_ values (µM) for **L1** and **L2** and the iron(II) cylinders against HCT116 (human colorectal carcinoma), NCI-H460 (human non-small cell lung carcinoma), SiHa (human cervical carcinoma), and SW480 (human colon adenocarcinoma) cancer cells as compared to cisplatin, expressed as mean ± standard error (*n* = 3, incubation period 72 h).

Compound	IC_50_/μM			
	HCT116	NCI-H460	SiHa	SW480
**L1**	n.d.	n.d.	n.d.	n.d.
**L2**	n.d.	n.d.	n.d.	n.d.
*rac*-[Fe_2_(**L1**)_3_](BF_4_)_4_	7.1 ± 0.5	4.9 ± 0.4	39 ± 2	24 ± 4
*rac/meso*-[Fe_2_(**L2**)_3_](BF_4_)_4_	19 ± 1	46 ± 15	55 ± 15	13 ± 4
**L**_**pyim**_ Steel et al. (2021a)	21 ± 6	31 ± 4	42 ± 3	46 ± 5
*rac*-[Fe_2_(**L**_**Pyim**_)_3_](BF_4_)_4_	9 ± 2	10 ± 3	26 ± 4	28 ± 7
[(Rh(Cp*)Cl)_2_(**L**_**pyim**_)] Steel et al. (2021a)	78 ± 21	>100	45 ± 4	73 ± 5
[Fe(H_2_O)_6_](BF_4_)_2_	>200	184 ± 11	>500	>300
Cisplatin Tremlett et al. (2019)	2.5 ± 0.3	0.80 ± 0.03	3.0 ± 0.6	8.1 ± 2.9

n.d., not determined due to the poor solubility of the compound.

## Conclusion

Two new di(2,2′-bipyridine) ligands, 2,6-bis([2,2′-bipyridin]-5-ylethynyl)-pyridine (**L1**) and bis(4-([2,2′-bipyridin]-5-ylethynyl)-phenyl)methane (**L2**) were synthesized and exploited to generate two triple-stranded metallo-cylinders, [Fe_2_(**L1**)_3_](BF_4_)_4_ and [Fe_2_(**L2**)_3_](BF_4_)_4_. The ligands and cylinders were characterized by elemental analysis, ESIMS and UV-vis, ^1^H−, ^13^C−, and DOSY-NMR spectroscopies. The molecular structures of the [Fe_2_ **L**_3_](BF_4_)_4_ cylinders were confirmed using X-ray crystallography. Both [Fe_2_(**L1**)_3_](BF_4_)_4_ and [Fe_2_(**L2**)_3_](BF_4_)_4_ crystallized as racemic (*rac*) mixtures of the ΔΔ (P) and ΛΛ (M) helicates. NMR spectroscopy and ESIMS confirmed the presence of the [Fe_2_ **L**_3_]^4+^ supramolecular architectures in solution. However, ^1^H NMR spectra showed that in solution the larger [Fe_2_(**L2**)_3_](BF_4_)_4_ was present as a mixture of the *rac*-ΔΔ/ΛΛ and *meso*-ΔΛ isomers. The host-guest chemistry of the helicates, which both feature an accessible central cavity, was examined with several small drug molecules, including cisplatin and 5-fluorouracil. However, none of the potential guests were found to bind within the helicates, despite molecular modelling confirming that there were no obvious steric impediments to the interaction. Cytotoxicity assays demonstrated that both helicates were active against the four cell lines examined. The smaller *rac*-[Fe_2_(**L1**)_3_](BF_4_)_4_ helicate was more cytotoxic than the larger *rac/meso*-[Fe_2_(**L2**)_3_](BF_4_)_4_ analogue and displayed promising low μM antiproliferative activity against HCT116 (IC_50_ = 7.1 ± 0.5 μM) and NCI-H460 (IC_50_ = 4.9 ± 0.4 μM) human cancer cells. Although both helicates were less active than the widely used anti-cancer drug cisplatin, these results suggest that helicates constructed from di(2,2′-bipyridine) ligands have potential as anti-cancer agents in their own right. The combination of a cytotoxic supramolecular structure with encapsulated drugs may result in synergistic activity. However, the poor aqueous solubility and modest stability in biological media of the current [Fe_2_(**L1**)_3_](BF_4_)_4_ helicates means that the properties of these compounds will need to be fine-tuned to overcome these shortfalls. This could potentially be achieved by using more kinetically inert metal ions such as Ru(II) (Glasson et al., 2008; Kumar et al., 2015) or Co(III) (Symmers et al., 2015; Burke et al., 2018; Crlikova et al., 2020) to assemble the helicates.

## Data Availability

The original contributions presented in the study are included in the article/Supplementary Material, further inquiries can be directed to the corresponding author.
