# Corrigendum: Cavity-Containing [Fe_2_L_3_]^4+^ Helicates: An Examination of Host-Guest Chemistry and Cytotoxicity

**DOI:** 10.3389/fchem.2021.739785

**Published:** 2021-08-03

**Authors:** Lynn S. Lisboa, Mie Riisom, Roan A. S. Vasdev, Stephen M. F. Jamieson, L. James Wright, Christian G. Hartinger, James D. Crowley

**Affiliations:** ^1^ Department of Chemistry, University of Otago, Dunedin, New Zealand; ^2^ School of Chemical Sciences, University of Auckland, Auckland, New Zealand; ^3^ Auckland Cancer Society Research Centre, University of Auckland, Auckland, New Zealand

**Keywords:** iron(II), helicate, cytotoxicity, host-guest chemistry, metallosupramolecular architectures

In the original article, there was a mistake in **Figure 1** as published. The N positions of the pyridines in the ligand structure are incorrect. The corrected **Figure 1** appears below.

The authors apologize for this error and state that this does not change the scientific conclusions of the article in any way. The original article has been updated.

**FIGURE 1:**
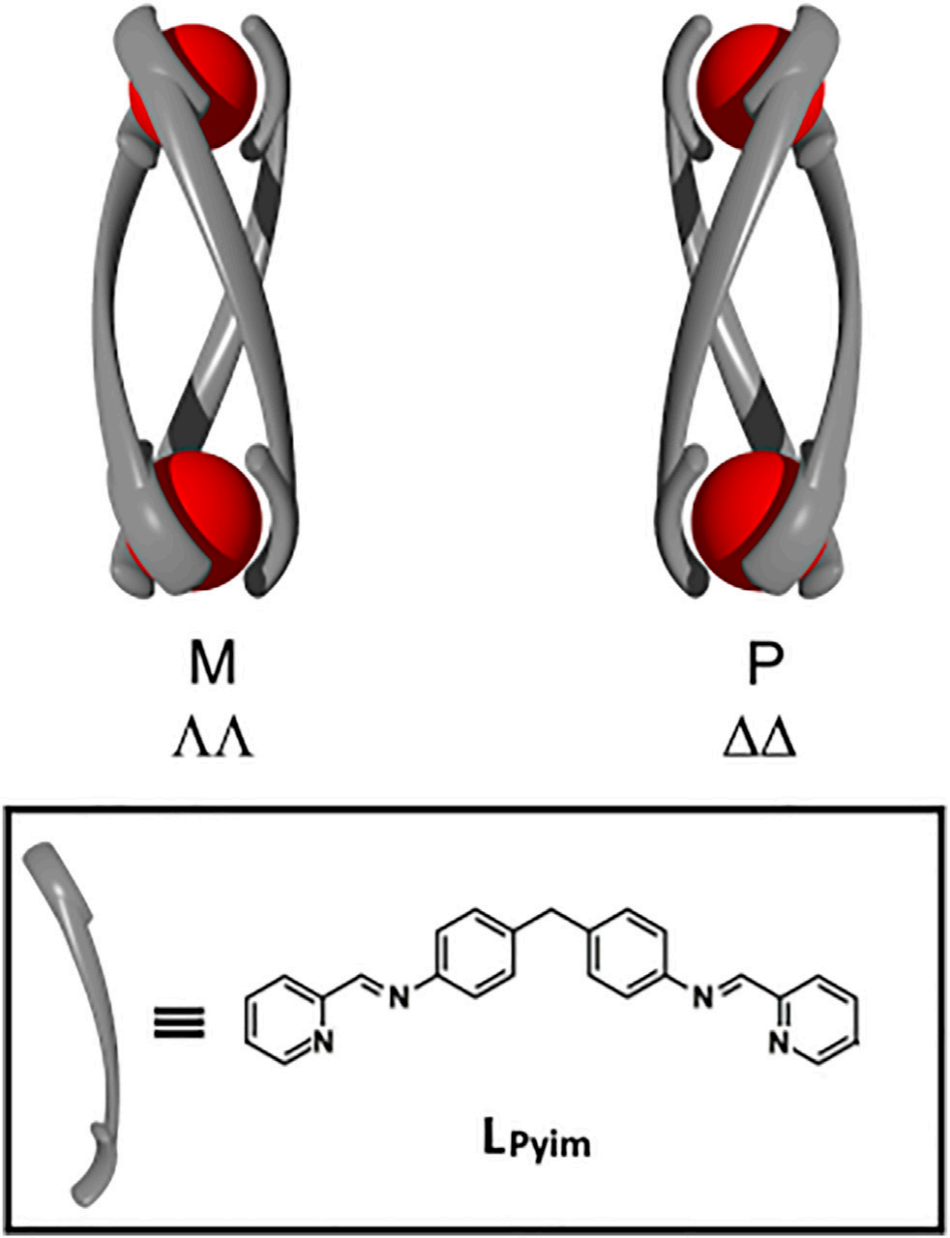
Cartoon representations of the minus (M, ΛΛ), plus (P, ΔΔ) helicate isomers of a generic triple-stranded helicate and the chemical structure of the (1E,1′E)-N,N′-[methylenebis(4,1-phenylene)]bis[1-(pyridin-2-yl)methanimine] ligand (**L**_**Pyim**_) developed by Hannon and co-workers (Hannon et al., 1997).

