# Supplementary material for: Cavity-Containing [Fe2L3]4+ Helicates: An Examination of Host-Guest Chemistry and Cytotoxicity
Source: Front Chem. 2021 Jul 7;9:697684. doi: 10.3389/fchem.2021.697684 (PMC8292671; doi:10.3389/fchem.2021.697684)
Supplement: Supplementary file 1 [file DataSheet1.docx]

Supporting Information

**Cavity-containing [Fe_2_L_3_]^4+^ helicates: An examination of Host-Guest Chemistry and Cytotoxicity.**

Lynn S. Lisboa^1^, Mie Riisom^2,3^, Roan A. S. Vasdev^1^, Stephen M. F. Jamieson^3^, L. James Wright^2^, Christian G. Hartinger^2^, James D. Crowley^1*^

^1^Department of Chemistry, University of Otago, PO Box 56, Dunedin, 9054 New Zealand

^2^School of Chemical Sciences, University of Auckland, Private Bag 92019, Auckland, 1142 New Zealand

^3^Auckland Cancer Society Research Centre, University of Auckland, Private Bag 92019, Auckland 1142, New Zealand

***jcrowley@chemistry.otago.ac.nz**

**Contents**

[1 Experimental Procedures 4](#_Toc73543475)

[1.1 General 4](#_Toc73543476)

[1.2 Synthetic Schemes 4](#_Toc73543477)

[1.2.1 Synthetic scheme for 5-ethynyl-2,2'-bipyridine, **1c** 4](#_Toc73543478)

[1.3 Experimental 5](#_Toc73543479)

[1.3.1 **L1** 5](#_Toc73543480)

[1.3.2 **L2** 6](#_Toc73543481)

[1.3.3 [Fe_2_(**L1**)_3_](BF_4_)_4_ 7](#_Toc73543482)

[1.3.4 [Fe_2_(**L2**)_3_](BF_4_)_4_ 9](#_Toc73543483)

[1.4 ^1^H Diffusion Ordered Spectroscopy (DOSY NMR) 11](#_Toc73543484)

[1.5 ^1^H NMR stability study 12](#_Toc73543485)

[1.6 UV-Visible spectroscopy 12](#_Toc73543486)

[1.7 UV-visible stability study 14](#_Toc73543487)

[1.8 Alternative counteranions (OTF^-^ and Cl^-^) 17](#_Toc73543488)

[1.8.1 General procedure for the synthesis of the helicates with OTf^-^ counteranions 17](#_Toc73543489)

[1.8.2 General procedure for the synthesis of the helicates with Cl^-^ counteranions 18](#_Toc73543490)

[1.9 ^19^F NMR Spectroscopy 19](#_Toc73543491)

[1.10 Guest-binding 19](#_Toc73543492)

[1.10.1 [Fe_2_(**L1**)_3_](BF_4_)_4_ 19](#_Toc73543493)

[1.10.2 [Fe_2_(**L2**)_3_](BF_4_)_4_ 20](#_Toc73543494)

[1.11 Host-Guest MMFF models 21](#_Toc73543495)

[2 Sulforhodamine B Cytotoxicity Assay. 22](#_Toc73543496)

[3 X-ray Crystallography 23](#_Toc73543497)

[Table 2 X-ray data tables 25](#_Toc73543498)

[Table 3 X-ray data tables 26](#_Toc73543499)

[4 References 27](#_Toc73543500)

# Experimental Procedures

## General

All reagents were purchased from commercial sources and used without further purification except 1,4-benzoquinone which was purified by passing through a silica plug with dichloromethane prior to use. TLC plates (200 µm thickness) and silica gel (40-63 µm) were purchased from Silicycle. 5-Bromo-2,2'-bipyridine (**1a**),(Kim and Rieke, 2010) 5-((trimethylsilyl)ethynyl)-2,2'-bipyridine (**1b**),(Grosshenny et al., 1997) 5-((trimethylsilyl)ethynyl)-2,2'-bipyridine (**1c**)(Grosshenny et al., 1997) and bis(4-iodophenyl)methane(Austin et al., 1981) were synthesized using previously reported methods.(Scottwell et al., 2015) ^1^H and ^13^C{^1^H} NMR spectra were recorded on either a 400 MHz Varian 400 MR or a Varian 500 MHz VNMRS spectrometer. ^1^H DOSY NMR spectra were obtained on a Varian 500 MHz VNMRS spectrometer and processed using the DOSY package on VNMRJ^®^ Version 4.2 software. Chemical shifts are reported in parts per million and referenced to residual solvent peaks (chloroform-*d*: ^1^H δ 7.26 ppm, ^13^C δ 77.16 ppm; acetonitrile-*d*_3_: ^1^H δ 1.94, ^13^C δ 1.32, 118.26 ppm; DMSO-*d*_6_: ^1^H δ 2.50, ^13^C δ 39.52). Coupling constants (*J*) are reported in Hertz (Hz). Standard abbreviations indicating multiplicity were used as follows: m = multiplet, s = singlet, d = doublet, t = triplet, q = quartet, quin = quintet, dd = doublet of doublets, dt = doublet of triplets, td = triplet of doublets. Other abbreviations include TEA = trimethylamine, THF = tetrahydrofuran, DMSO = dimethylsulfoxide, DCM = dichloromethane. Microanalyses were performed at the Campbell Microanalytical Laboratory at the University of Otago. Electrospray ionization mass spectra (ESIMS) were collected on a Bruker micro-TOF-Q spectrometer; spectra for [Fe_2_(**L1**)_3_](BF_4_)_4_ and [Fe_2_(**L2**)_3_](BF_4_)_4_ were obtained using coldspray conditions (nebuliser and heating gasses cooled to -10 °C). UV-visible absorption spectra were acquired with a Shimadzu UV-2600 spectrophotometer.

## Synthetic Schemes

### Synthetic scheme for 5-ethynyl-2,2'-bipyridine, 1c

Scheme S1 Synthesis of 5-((trimethylsilyl)ethynyl)-2,2'-bipyridine (1c):(i) [Pd(PPh_3_)_4_], dry THF, reflux, 5 hours (ii) ethynyltrimethylsilane, CuI, [Pd(PPh_3_)_2_Cl_2_], TEA, THF, 65 °C, 16 hours (iii) Na_2_CO_3_, MeOH, RT, 1 hour.

## Experimental

### L1

**1c** (0.50 g, 2.8 mmol) and 2,6-dibromopyridine (0.30 g, 1.3 mmol) were added to degassed TEA:THF (1:3 v/v, 25 mL) under N_2(g)_. Copper(I) iodide (0.024 g, 0.13 mmol) and bis(triphenylphosphine)palladium(II) dichloride (0.022 g, 0.032 mmol) were added to the solution. The solution was heated at 65 °C for 16 hours. The solvents were removed under vacuum to give the crude product (brown oil) The crude product was dissolved in dichloromethane (50 mL) before washing with EDTA/NH_4_OH (0.10 M, 50 mL), water (50 mL) and brine (50 mL). The product was partially purified by column chromatography (silica gel deactivated with 5% TEA/DCM, acetone). The fractions containing the product (R_f_ = 0.67, deactivated silica plates, 1:8 acetone:DCM) were collected, combined and dried under vacuum. The solid residue was suspended in acetone (5 mL) and the product was collected by filtration to afford pure **L1** as an off-white solid (0.280 g, 51%). ^1^H NMR (400 MHz, CDCl_3_) δ 8.89 (s, 2H_g_), 8.71 (d, *J* = 4.3 Hz, 2H_a_), 8.52 – 8.40 (m, 4H_d,e_), 8.02 (dd, *J* = 8.3, 2.1 Hz, 2H_f_), 7.85 (td, *J* = 7.7 Hz, 2H_c_), 7.76 (t, 1H_i_), 7.57 (d, *J* = 7.8 Hz, 2H_h_), 7.39 – 7.31 (m, 2H_b_); ^13^C{^1^H} NMR (100 MHz, CDCl_3_) δ 155.5, 155.2, 152.1, 149.2, 143.4, 139.9, 137.1, 136.7, 126.8, 124.1, 121.6, 120.5, 119.1, 92.1, 86.8; ESIMS: (CHCl_3_) *m/z* = 436.1545 (calc. for [**L1** + H]^+^ 436.1557 m/z), 458.1372 (calc. for [**L1** + Na]^+^: 458.1372 m/z); Anal. Calc. for C_29_H_17_N_5_⋅1.4H_2_O: C 75.72; H 4.00; N 14.90%. Found C 75.61; H 4.33; N 14.20%.


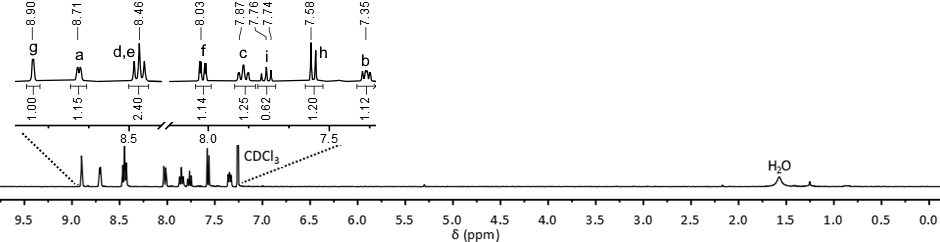


Figure S1 ^1^H NMR spectrum (400 MHz, 298 K, CDCl_3_) of L1


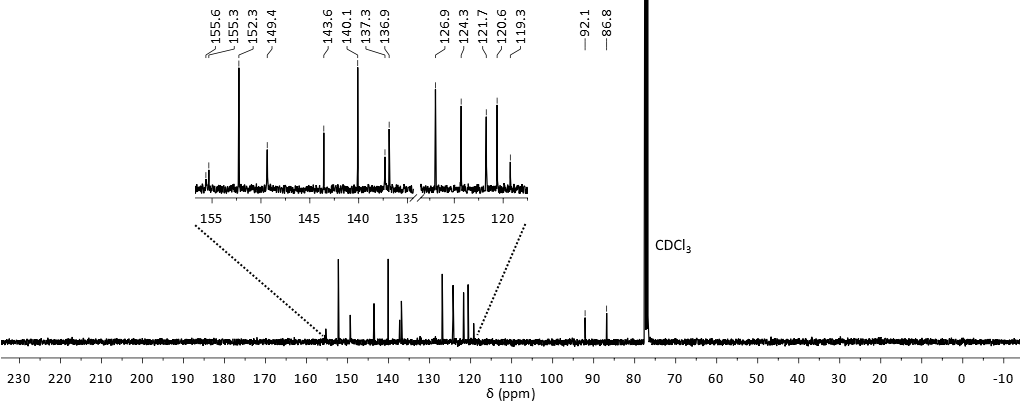


Figure S2 ^13^C{^1^H} NMR spectrum (100 MHz, 298 K, CDCl_3_) of L1

### L2

**1c** (0.53 g, 2.9 mmol) and bis(4-iodophenyl)methane (0.56 g, 1.3 mmol) were added to degassed TEA:THF (1:3 v/v, 26 mL) under N_2(g)_. Copper(I) iodide (0.030 g, 0.13 mmol) and bis(triphenylphosphine)palladium(II) dichloride (0.020 g, 0.030 mmol) were then added and the resulting mixture was heated at 65 °C for 16 hours. The solvents were removed under vacuum to yield a crude product mixture (brown oil). The crude mixture was dissolved in chloroform (75 mL) before washing with EDTA/NH_4_OH (0.10 M (75 mL), water (75 mL) and brine (75 mL). The product was partially purified by column chromatography (silica gel deactivated with 5% TEA/ DCM, 1:1 DCM:acetone). Fractions containing the product (R_f_ = 0.41, deactivated silica plates, DCM) were dried under vacuum and suspended in dichloromethane (5 mL). The colorless solid was collected by filtration to afford pure **L2** (0.43 g, 61%). ^1^H NMR (400 MHz, CDCl_3_) δ 8.81 (d, *J* = 2.0, 0.9 Hz, 2H, H_g_), 8.69 (dd, *J* = 4.7, 1.8, 0.9 Hz, 2H, H_a_), 8.46 – 8.39 (m, 4H, H_d,e_), 7.93 (dd, *J* = 8.3, 2.1 Hz, 2H, H_f_), 7.84 (td, *J* = 7.8, 1.8 Hz, 2H, H_c_), 7.53 – 7.49 (m, 4H, H_h_), 7.33 (dd, *J* = 7.5, 4.8, 1.2 Hz, 2H, H_b_), 7.22 – 7.19 (m, 4H, H_i_), 4.04 (s, 2H, H_j_); ^13^C NMR (100 MHz, CDCl_3_) δ 155.4, 154.7, 151.6, 149.2, 141.4, 139.3 137.0, 131.9, 129.1, 123.9, 121.4, 120.6, 120.4, 120.3, 93.6, 86.4, 41.9; ESIMS: (chloroform) *m/z* = 525.2037 (calc. for [**L2** + H]^+^: 525.2074 m/z), 547.1869 (calc. for [**L2** + Na]^+^: 547.1893 m/z); Anal. Calc. for C_37_H_24_N_4_⋅0.4CHCl_3_: C 78.48; H 4.30; N 9.79%. Found C_37_H_24_N_4_: C 78.17; H 4.84; N 10.22%.


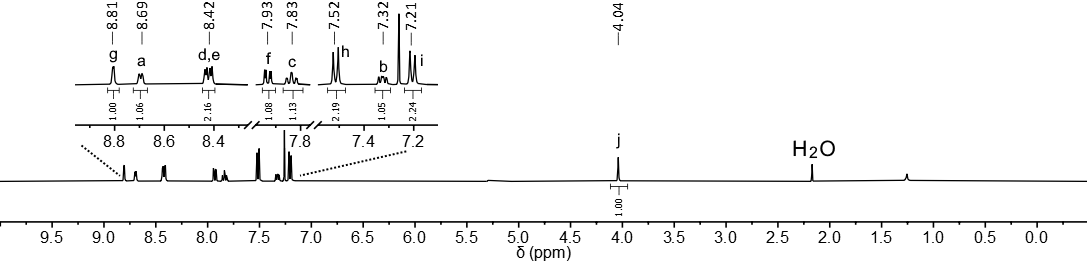


Figure S3 ^1^H NMR spectrum (400 MHz, 298 K, CDCl_3_) of L2


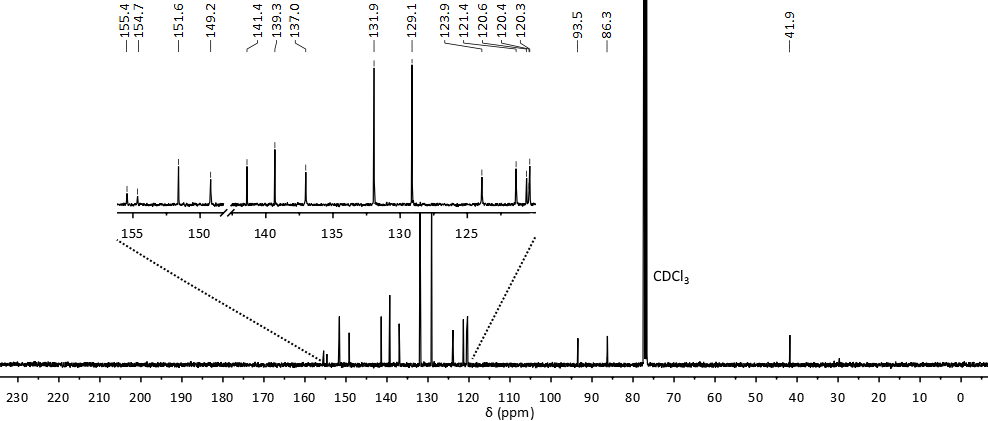


Figure S4 ^13^C{^1^H} NMR spectrum (100 MHz, 298 K, CDCl_3_) of L2

### [Fe_2_(L1)_3_](BF_4_)_4_

2,6-Bis([2,2'-bipyridin]-5-ylethynyl)pyridine (**L1**) (0.020 g, 0.046 mmol) and iron(II) tetrafluoroborate hexahydrate ([Fe(H_2_O)_6_](BF_4_)_2_) (0.012 g, 0.031 mmol) were combined in acetonitrile (5 mL) and stirred at 65 °C for 16 hours. The solvent was removed from the reaction mixture under vacuum and the resulting residue suspended in chloroform (5 mL). The dark red solid was collected by centrifugation. The pellet was further washed with ether and centrifuged again. The pellet was dissolved in nitromethane (1 mL) and crystals of [Fe_2_(**L1**)_3_](BF_4_)_4_ were obtained by slow vapor diffusion of diethyl ether into the solution. The purified [Fe_2_(**L1**)_3_](BF_4_)_4_ was collected as dark red needle-shaped crystals and washed with diethyl ether (0.024 g, 89%). ^1^H NMR (500 MHz, CD_3_CN) δ 8.53 (t, *J* = 7.7 Hz, 12H, H_d,e_), 8.20 (d, *J* = 8.4, 1.8 Hz, 6H, H_f_), 8.15 (t, *J* = 7.6, 2.5 Hz, 6H, H_c_), 7.83 (t, *J* = 7.9 Hz, 3H, H_i_), 7.72 (s, 6H, H_g_), 7.56 (d, *J* = 7.9 Hz, 6H, H_h_), 7.43 (t, *J* = 6.7 Hz, 6H, H_b_), 7.27 (d, *J* = 5.6 Hz, 6H, H_a_). ^13^C{^1^H} NMR (125 MHz, CD_3_CN) δ 159.69, 159.40, 157.98, 155.23, 143.23, 142.12, 139.94, 138.83, 128.94, 125.82, 124.56, 123.09, 94.36, 84.08. ESIMS: *m/z* = 354.5886 (calc. for [Fe_2_(**L1**)_3_]^4+^: 354.5791), Anal. Calc. for C_87_H_51_B_4_F_16_Fe_2_N_15_: C 59.91; H 2.91; N 11.90%. Found: C 58.79; H 2.82; N 11.95%.


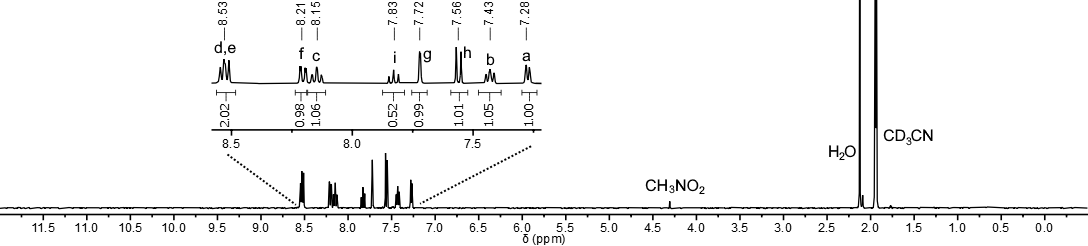


Figure S5 ^1^H NMR spectrum (500 MHz, 298 K, CD_3_CN) of [Fe_2_(L1)_3_](BF_4_)_4_.


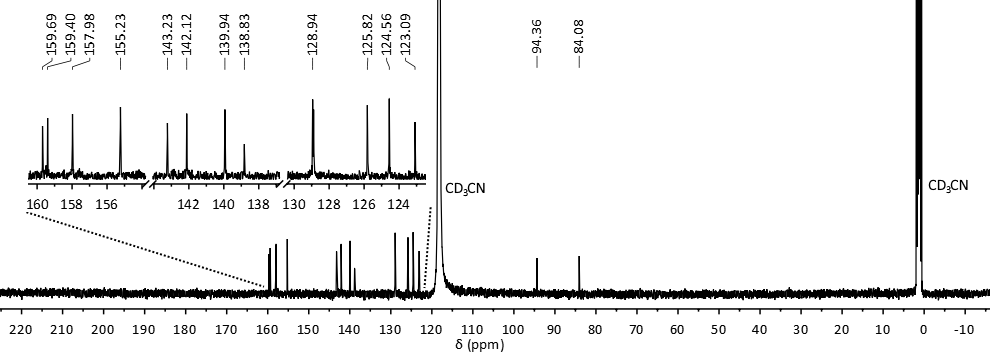


Figure S6 ^13^C{^1^H} NMR spectrum (125 MHz, 298 K, CD_3_CN) of [Fe_2_(L1)_3_](BF_4_)_4_.


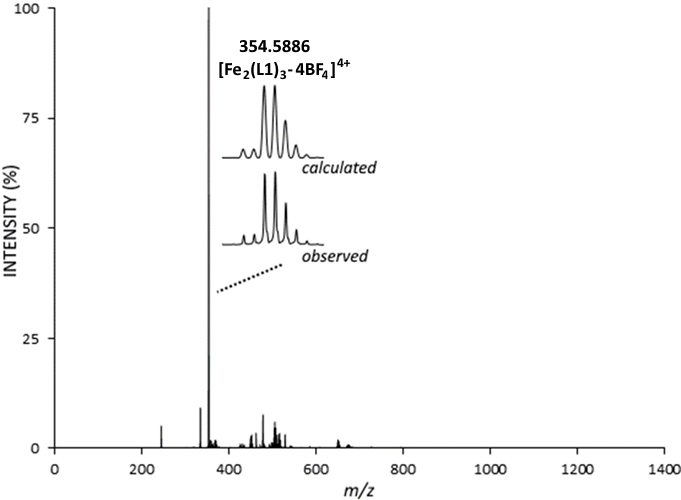


Figure S7 Electrospray ionisation mass spectrum (CH_3_CN) of [Fe_2_(L1)_3_](BF_4_)_4_ performed under pseudo-coldspray conditions.

### [Fe_2_(L2)_3_](BF_4_)_4_

Bis(4-([2,2'-bipyridin]-5-ylethynyl)phenyl)methane (**L2**) (0.020 g, 0.038 mmol) and iron(II) tetrafluoroborate hexahydrate (0.009 g, 0.03 mmol) were added to acetonitrile (5 mL). The dark red solution was stirred at 65 °C for 16 hours. The solvent was removed under vacuum to provide a crude dark red product. This material was suspended in chloroform and the dark red solid was collected by centrifugation. The pellet was further washed with ether and centrifuged again. The dark red pellet was dissolved in nitromethane (1 mL) and crystals of [Fe_2_(**L2**)_3_](BF_4_)_4_ were obtained by slow vapor diffusion of diethyl ether into the solution. The purified [Fe_2_(**L2**)_3_](BF_4_)_4_ was collected as dark red cubic crystals and washed with diethyl ether (0.020 g, 77%). ^1^H NMR (500 MHz, CD_3_CN) δ 8.58 – 8.50 (m, 6H, H_d,e_), 8.23 – 8.11 (m, 6H, H_c,f_), 7.61 – 7.53 (m, 3H, H_g_), 7.45 – 7.21 (m, 18H, H_a,b,h,i_), 4.05 – 3.84 (m, 6H, H_j_). ^13^C{^1^H} NMR (125 MHz, CD_3_CN) δ 159.65, 158.77, 156.92, 155.22, 144.35, 141.85, 139.94, 133.04, 130.30, 128.69, 125.57, 124.82, 124.64, 120.20, 97.27, 85.19, 42.32. The broadness of the peaks in the ^1^H NMR spectrum, splitting of j and the doubling of ^13^C NMR peaks is indicative of a mixture of ΔΛ and ΛΛ/ΔΔ isomers. ESIMS: *m/z* = 421.3972, (calc. for [Fe_2_(**L2**)_3_]^4+^: 421.3678). Despite multiple attempts to collect elemental analysis, satisfactory data were not obtained presumably due to the large amount of solvents that are contained in the crystals.


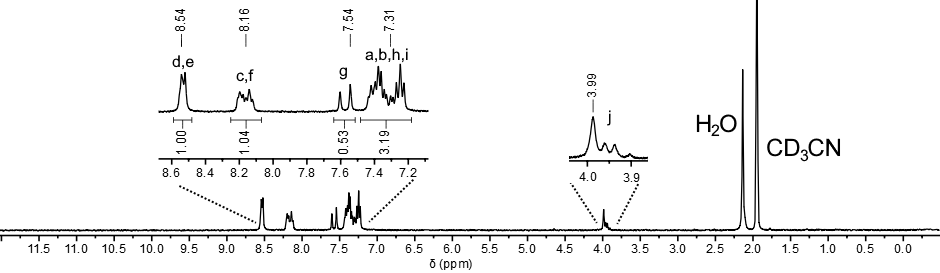


Figure S8 ^1^H NMR (400 MHz, 298 K, CD_3_CN) of [Fe_2_(L2)_3_](BF_4_)_4_. Note: The peaks in the aromatic region appear broad suggestive of a mixture of the *meso*-ΔΛ and *rac-*ΛΛ/ΔΔ isomers. The splitting of the methylene resonance (H_j_ δ = 4.0-3.9 ppm) into a singlet and a doublet of doublets confirms this.


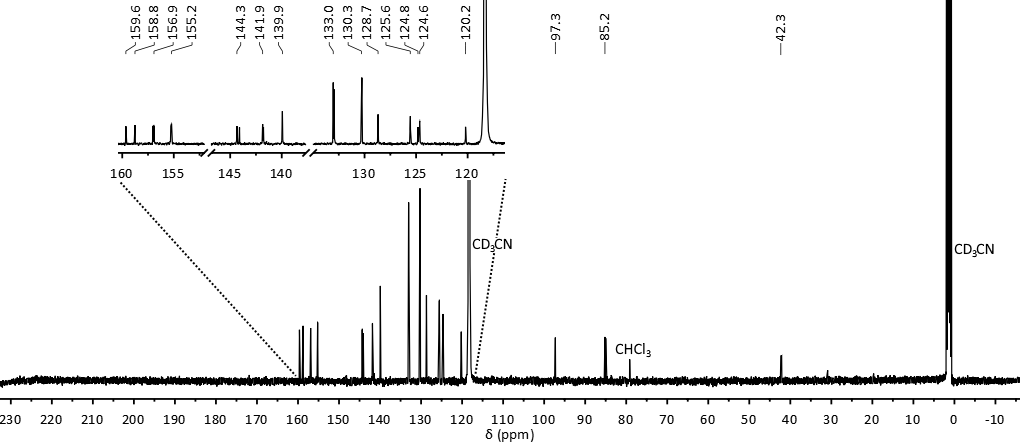


Figure S9 ^13^C{^1^H} NMR spectrum (125 MHz, 298 K, CD_3_CN) of [Fe_2_(L2)_3_](BF_4_)_4_. Note: most peaks appear in doubles due to the compound being a mixture of ΔΛ and ΛΛ/ΔΔ isomers.


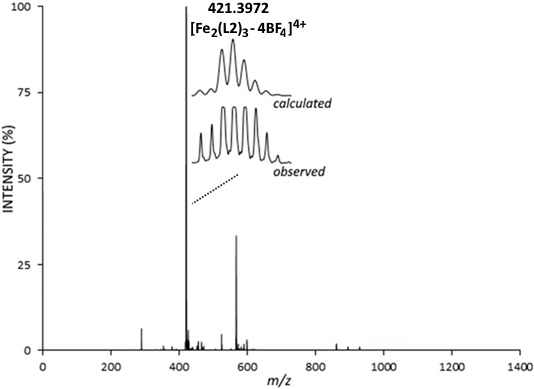


Figure S10 Electrospray ionisation mass spectrum (acetonitrile) of [Fe_2_(L2)_3_](BF_4_)_4_ performed under coldspray conditions. Note: The observed peak for [Fe_2_(L2)_3_]^3+^ is truncated due to the high concentration required to obtain a good signal.

## ^1^H Diffusion Ordered Spectroscopy (DOSY NMR)

Table 1 Diffusion coefficients as obtained via ^1^H DOSY NMR experiments (500 MHz, CD_3_CN_,_ 298 K).

| **Species** | **Molecular Weight (gmol^-1^)** | **Diffusion Coefficient (x 10^-10^ m^2^s^-1^)** |
| --- | --- | --- |
| [Fe_2_(**L1**)_3_](BF_4_)_4_ | 1765.34 | 6.51 ± 0.03 |
| [Fe_2_(**L2**)_3_](BF_4_)_4_ | 2032.75 | 5.40 ± 0.10 |


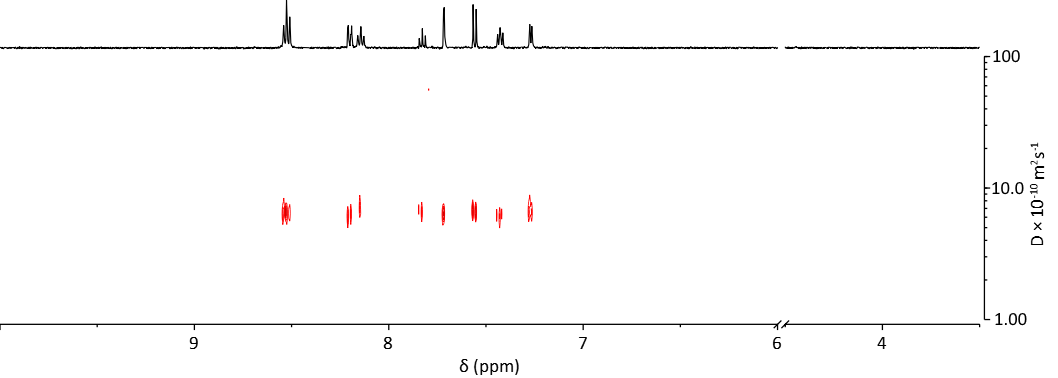


Figure S11 Partial ^1^H NMR and the corresponding DOSY NMR spectra (500 MHz, CD_3_CN, 298 K) of [Fe_2_(L1)_3_](BF_4_)_4_


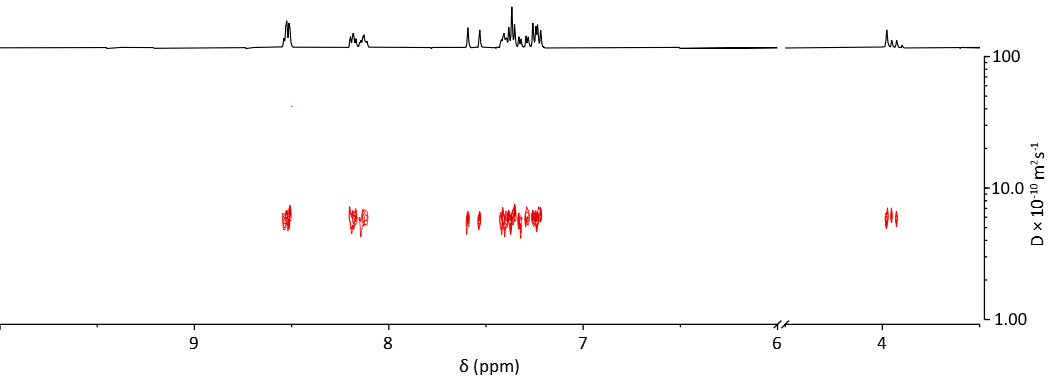


Figure S12 Partial ^1^H NMR and the corresponding DOSY NMR spectra (500 MHz, CD_3_CN, 298 K) of [Fe_2_(L2)_3_](BF_4_)_4_.

## ^1^H NMR stability study

^1^H NMR spectroscopic stability studies were carried out for [Fe_2_(**L1**)_3_](BF_4_)_4_ and [Fe_2_(**L2**)_3_](BF_4_)_4_ in a neat DMSO-*d*_6_ solution (1.0 × 10^-4^ mol L^-1^) with *tert*-butanol (0.060 mg, 0.81 µmol) as a standard. Attempts were made to collect ^1^H NMR data in a 1:19 DMSO-*d*_6_/D_2_O solution mixture (1.0 × 10^-3^ mol L^-1^) but spectra appeared broad and undefined.


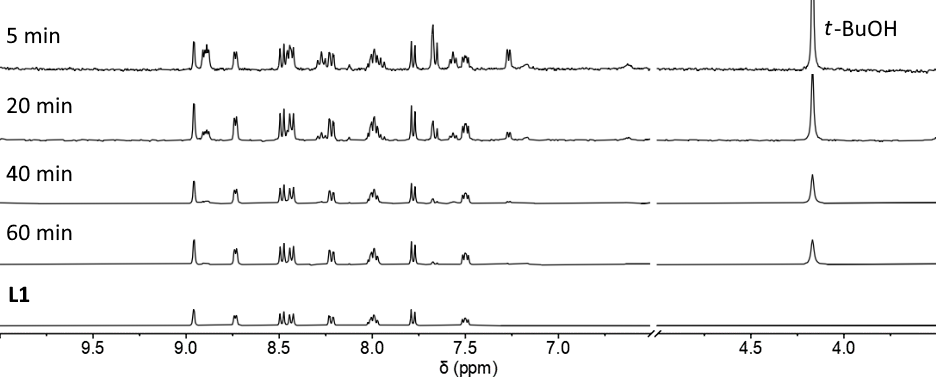


Figure S13 Stacked partial ^1^H NMR spectra (400 MHz, 298 K, DMSO-*d*_6_) showing the decomposition of [Fe_2_(L1)_3_](BF_4_)_4_ over time in DMSO.


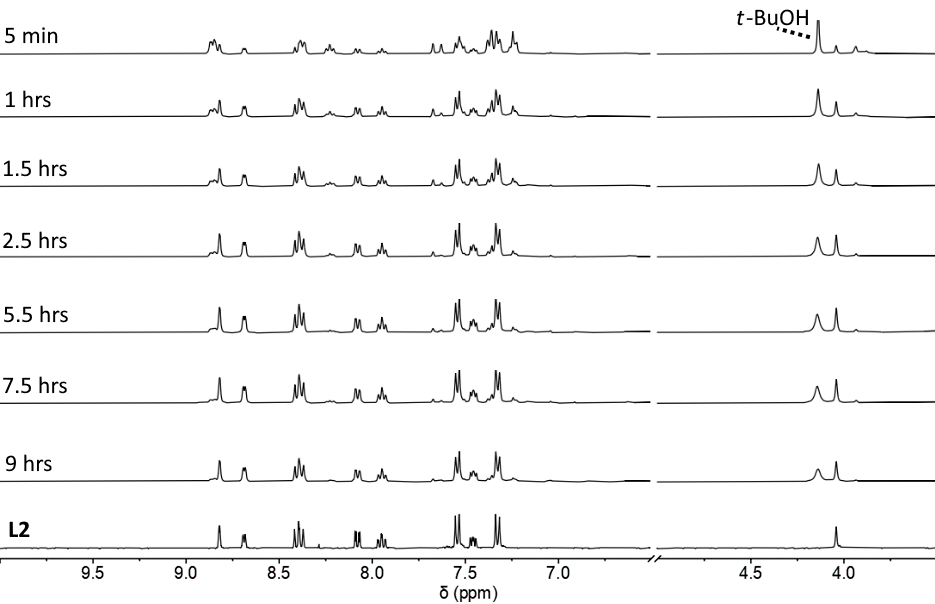


Figure S14 Stacked partial ^1^H NMR spectra (400 MHz, 298 K, DMSO-*d*_6_) showing the decomposition of [Fe_2_(L2)_3_](BF_4_)_4_ over time in DMSO.


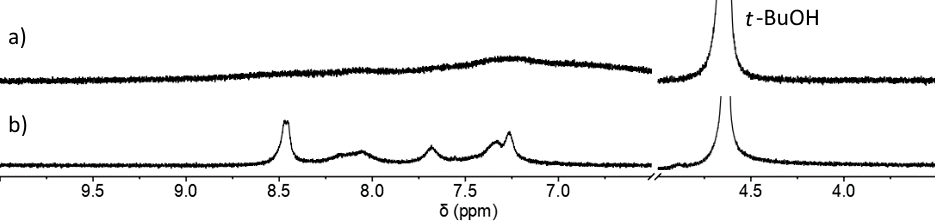


Figure S15 Stacked partial ^1^H NMR spectra (400 MHz, 298 K, D_2_O) showing the decomposition of a) [Fe_2_(L1)_3_](BF_4_) b) [Fe_2_(L2)_3_](BF_4_)_4_ over time in 1:19 DMSO/D_2_O.

## UV-Visible spectroscopy

UV-Vis absorption spectroscopy studies were carried out at a concentration of
1.0 × 10^-5^ mol L^-1^ in acetonitrile using quartz cuvettes.


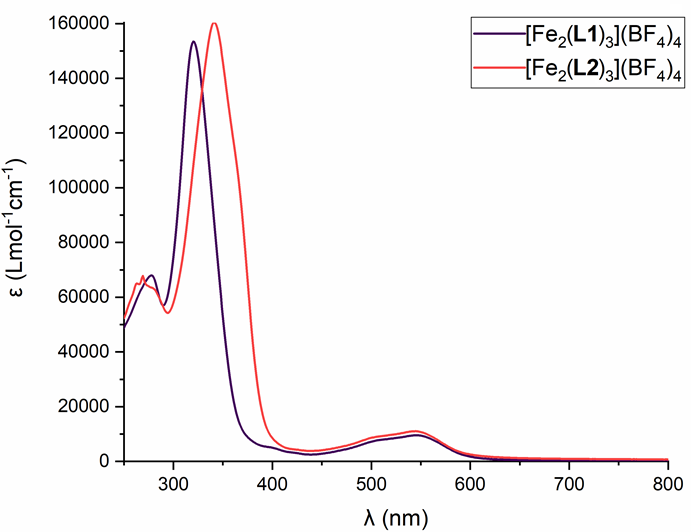


Figure S16 UV-visible absorption spectra (CH_3_CN, 1.0 × 10^-5^ mol L^-1^) of [Fe_2_(L1)_3_](BF_4_)_4_ (purple) and [Fe_2_(L2)_3_](BF_4_)_4_ (red)

## UV-visible stability study

UV-visible spectroscopic stability studies were carried out for [Fe_2_(**L1**)_3_](BF_4_)_4_ and [Fe_2_(**L2**)_3_](BF_4_)_4_ in a neat DMSO solution (1.0 × 10^-4^ mol L^-1^) and a 1:19 v/v DMSO:water solution (1.0 × 10^-4^ mol L^-1^) using quartz cuvettes. In order to replicate the timeframe of the biological studies, 1:19 v/v DMSO:water studies were collected at time intervals over 72 hours.





Figure S17 The change in UV-visible absorption spectra of [Fe_2_(L1)_3_](BF_4_)_4_ over time DMSO


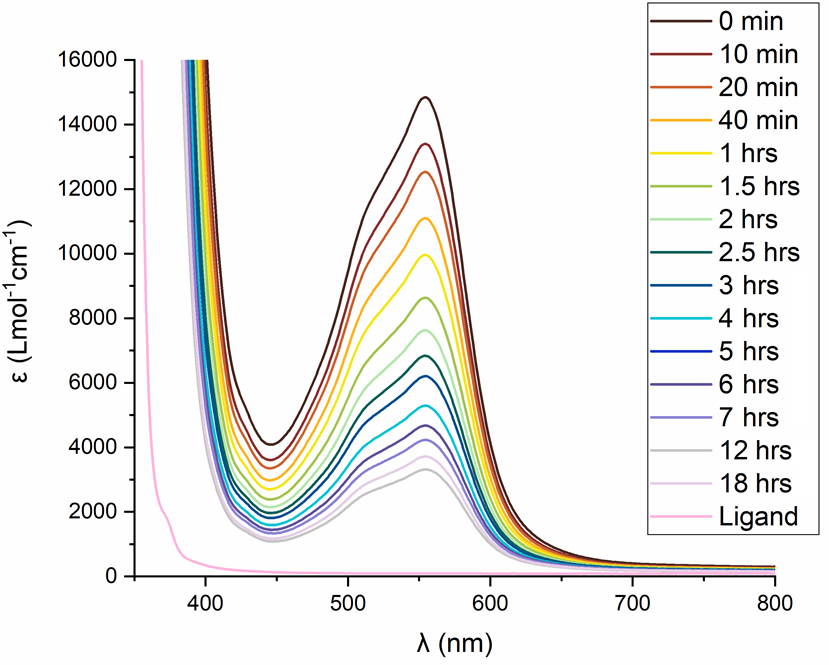


Figure S18 The change in UV-visible absorption spectra of [Fe_2_(L2)_3_](BF_4_)_4_ over time DMSO





Figure S19 The change in UV-visible absorption spectra of [Fe_2_(L1)_3_](BF_4_)_4_ over time in a solution of 1:19 v/v DMSO:water.


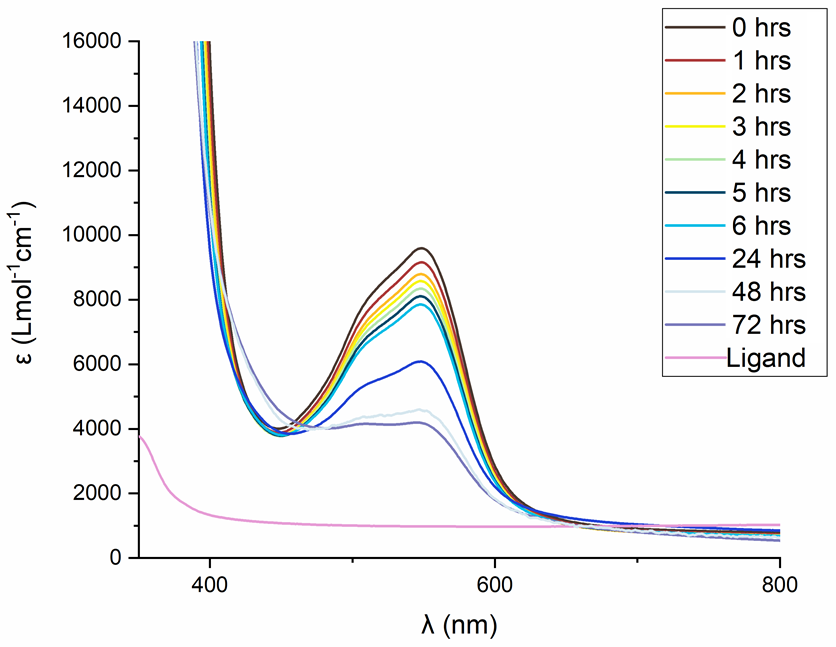


Figure S20 The change in UV-visible absorption spectra of [Fe_2_(L2)_3_](BF_4_)_4_ over time in a solution of 1:19 v/v DMSO:water.

## Alternative counteranions (OTF^-^ and Cl^-^)

For the purpose of biological testing, the water solubility of [Fe_2_(**L1**)_3_]^4+^ and [Fe_2_(**L2**)_3_]^4+^ with BF_4_^-^, OTf^-^ and Cl^-^ counteranions was tested. Neither of these complexes were sufficiently water soluble for biological testing. Therefore, biological testing was conducted using the BF_4_^-^ counteranions which appeared to be more soluble and stable for longer in DMSO.

### General procedure for the synthesis of the helicates with OTf^-^ counteranions

FeCl_2_ (0.013 mmol) and AgOTf (0.027 mmol) were combined in acetonitrile (0.50 mL) and stirred at room temperature for 1 hour. The resulting Fe(OTf)_2_ intermediate was centrifuged to remove AgCl_(s)_, and the supernatant was added to the ligand (**L1** or **L2**, 0.020 mmol). The AgCl pellet was washed with acetonitrile (0.50 mL), centrifuged, and the supernatant was again added to the reaction mixture. The resulting reaction mixture was heated at 65 °C for 16 hours. The acetonitrile was removed under vacuum and the crude mixture was suspended in dichloromethane (1.5 mL). This suspension was centrifuged and the supernatant discarded (x 3) and the product was obtained as a dark pink solid ([Fe_2_(**L1**)_3_](OTf)_4_, 9 mg, 73%; [Fe_2_(**L2**)_3_](OTf)_4_, 9 mg, 60%). ^1^H NMR spectra were collected in *d*_6_-DMSO (Figure S21 and Figure S22) and CD_3_CN (Figure S19 and Figure S20). [Fe_2_(**L1**)_3_](OTf)_4_ (^1^H NMR (400 MHz, CD_3_CN) δ 8.53 (dd, 8.72, 5.66 12H), 8.21 (d, *J* = 8.5 Hz, 6H), 8.14 (t, *J* = 7.8 Hz, 6H), 7.83 (t, *J* = 8.0 Hz, 3H), 7.72 (d, *J* = 8.2 Hz, 6H), 7.55 (d, *J* = 8.0 Hz, 6H), 7.43 (t, *J* = 6.3 Hz, 6H), 7.28 (d, *J* = 5.8 Hz, 6H)). [Fe_2_(**L2**)_3_](OTf)_4_ ^1^H NMR (400 MHz, CD_3_CN) δ 8.56 – 8.49 (m, 1H), 8.22 – 8.09 (m, 1H), 7.61 – 7.52 (m, 1H), 7.44 – 7.18 (m, 3H), 4.00 – 3.88 (m, 1H).





Figure S21 Stacked partial ^1^H NMR spectra (500 MHz, 298 K, CD_3_CN) of a) [Fe_2_(L1)_3_](BF_4_)_4_ and b) [Fe_2_(L1)_3_](OTf)_4_.





Figure S22 Stacked partial ^1^H NMR spectra (500 MHz, 298 K, CD_3_CN) of a) [Fe_2_(L2)_3_](BF_4_)_4_ and b) [Fe_2_(L2)_3_](OTf)_4_.

### General procedure for the synthesis of the helicates with Cl^-^ counteranions

**L1** or **L2** (0.020 mmol) and FeCl_2_ (0.013 mmol) were combined in acetonitrile (1 mL) and heated at 65 °C for 16 hours. The solvent was removed under vacuum and the crude solid was suspended in dichloromethane (1.5 mL). The suspension was centrifuged and the supernatant discarded (x 3). The final product was obtained as a dark pink solid ([Fe_2_(**L1**)_3_](Cl)_4_, 2 mg, 24% or [Fe_2_(**L2**)_3_](Cl)_4_, 6 mg, 50%). The resulting products were insoluble in acetonitrile and ^1^H NMR spectra (400 MHz, *d*_6_-DMSO) were collected within three minutes of sample preparation (Figure S21 and Figure S22). The spectra appeared broad with the presence of uncoordinated ligand, presumably due to the relative insolubility of these compounds and rapid ligand dissociation in DMSO.





Figure S23 Stacked partial ^1^H NMR spectra (500 MHz, 298 K, DMSO-*d*_6_) of a) L1, b) [Fe_2_(L1)_3_](BF_4_)_4_, c) [Fe_2_(L1)_3_](OTf)_4_ and d) [Fe_2_(L1)_3_](Cl)_4_.





Figure S24 Stacked partial ^1^H NMR spectra (500 MHz, 298 K, DMSO-*d*_6_) of a) L2, b) [Fe_2_(L2)_3_](BF_4_)_4_, c) [Fe_2_(L2)_3_](OTf)_4_ and d) [Fe_2_(L2)_3_](Cl)_4_.

## ^19^F NMR Spectroscopy





Partial ^19^F NMR spectra (376 MHz, 298 K, CD_3_CN) of [Fe_2_(L1)_3_](BF_4_)_4_, [Fe_2_(L2)_3_](BF_4_)_4_ and NaBF_4_ indicating association of the BF_4_ – counterion.


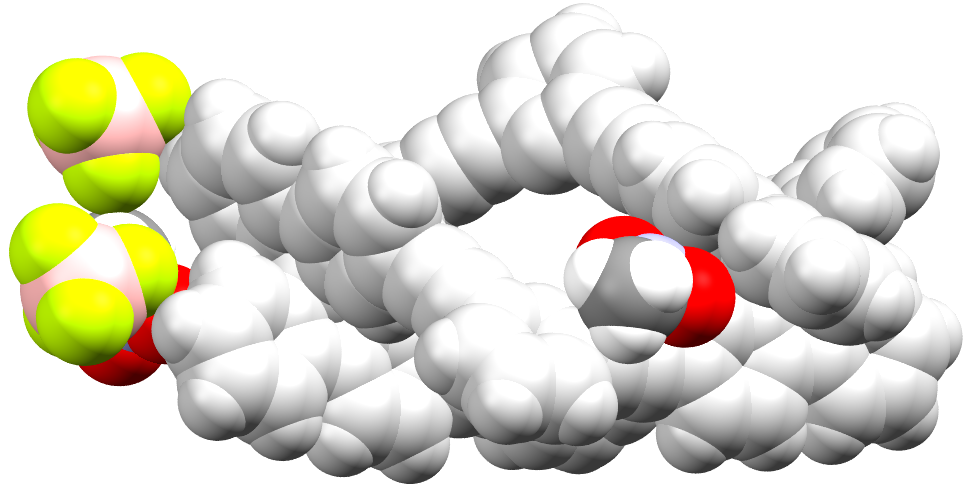


Figure S25 Highlighted nitromethane solvent molecules and BF_4_^-^ anions bound within the X-ray structure of [Fe_2_(L1)_3_](BF_4_)_4_.


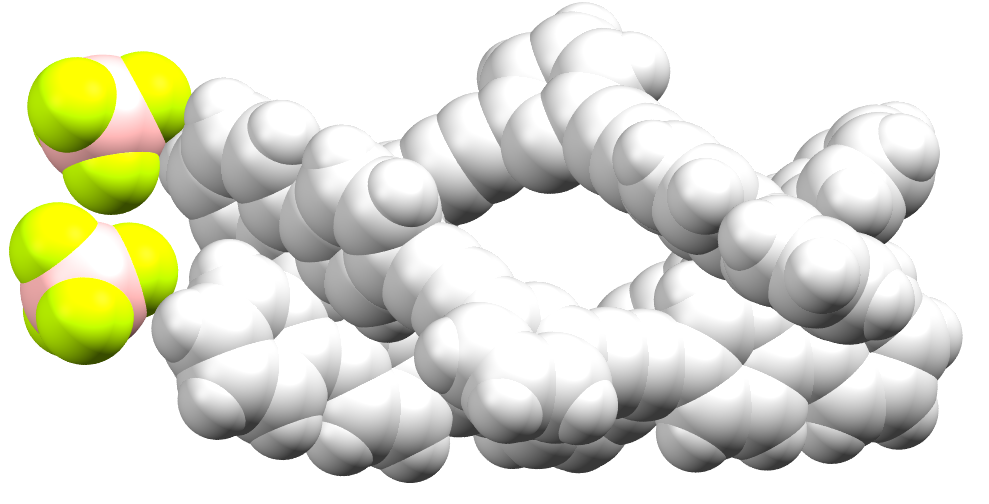


Figure S26 Highlighted BF_4_^-^ anions bound within the X-ray structure of [Fe_2_(L1)_3_](BF_4_)_4_.


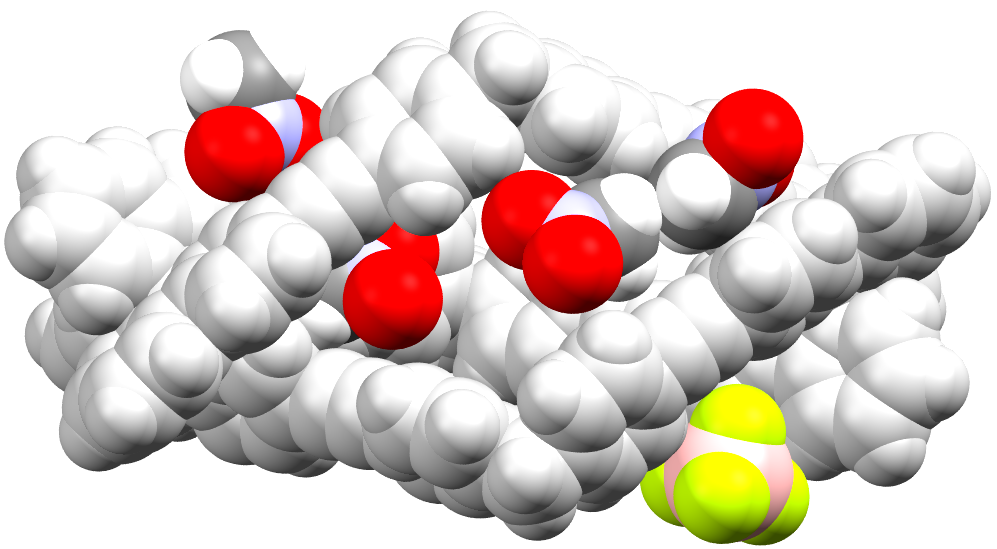


Figure S27 Highlighted nitromethane solvent molecules and BF_4_^-^ anions bound within the X-ray structure of [Fe_2_(L2)_3_](BF_4_)_4_.


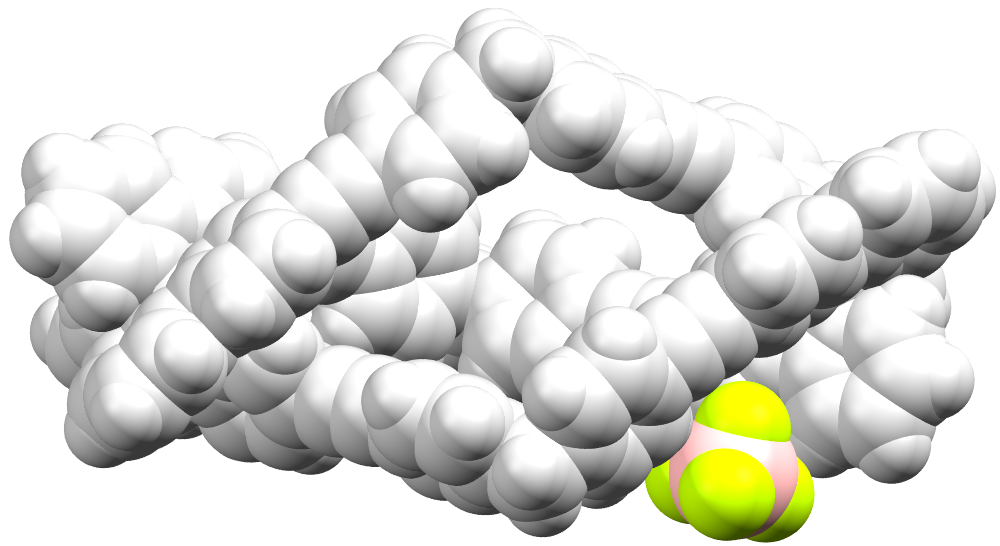


Figure S28 Highlighted BF_4_^-^ anions bound within the X-ray structure of [Fe_2_(L2)_3_](BF_4_)_4_.

## Guest-binding

Binding studies of the potential guest molecules 1,4-benzoquinone, 5-fluorouracil, acridine, nalidixic acid and cisplatin (Figure S25) within [Fe_2_(**L1**)_3_](BF_4_)_4_ and [Fe_2_(**L2**)_3_](BF_4_)_4_ were conducted at a 1:2 host:guest ratio in CD_3_CN. The potential guest molecules (1.1 µmol) were added as solids to a solution of [Fe_2_(**L1**)_3_](BF_4_)_4_ (0.57 µmol, 0.81 mM) and a ^1^H NMR spectrum (400 MHz, 298 K) was measured. The spectrum of the mixture was compared to that of the host and the guest to check for a shift in peaks, suggestive of guest binding. Similarly, the guest binding within [Fe_2_(**L2**)_3_](BF_4_)_4_ was studied with the potential guest molecules (0.98 µmol) being added as solids to solutions of [Fe_2_(**L2**)_3_](BF_4_)_4_ (0.49 µmol, 0.70 mM) in CD_3_CN.





Figure S29 Chemdraw structures of chosen guest molecules.

### [Fe_2_(L1)_3_](BF_4_)_4_





Figure S30 Stacked partial ^1^H NMR spectra (400 MHz, 298 K, CD_3_CN) of a) [Fe_2_(L1)_3_](BF_4_)_4_, b) [Fe_2_(L1)_3_](BF_4_)_4_ with 1,4-benzoquinone, c) [Fe_2_(L1)_3_](BF_4_)_4_ with 5-fluorouracil, d) [Fe_2_(L1)_3_](BF_4_)_4_ with nalidixic acid, e) [Fe_2_(L1)_3_](BF_4_)_4_ with acridine, f) [Fe_2_(L1)_3_](BF_4_)_4_ with cisplatin, g) 1,4-benzoquinone, h) 5-fluorouracil, i) nalidixic acid and j) acridine.

### [Fe_2_(L2)_3_](BF_4_)_4_





Figure S31 Stacked partial ^1^H NMR spectra (400 MHz, 298 K, CD_3_CN) of a) [Fe_2_(L2)_3_](BF_4_)_4_, b) [Fe_2_(L2)_3_](BF_4_)_4_ with 1,4-benzoquinone, c) [Fe_2_(L2)_3_](BF_4_)_4_ with 5-fluorouracil, d) [Fe_2_(L2)_3_](BF_4_)_4_ with nalidixic acid, e) [Fe_2_(L2)_3_](BF_4_)_4_ with acridine, f) [Fe_2_(L2)_3_](BF_4_)_4_ with cisplatin, g) 1,4-benzoquinone, h) 5-fluorouracil, i) nalidixic acid and j) acridine.

## Host-Guest MMFF models

All MMFF models were obtained using SPARTAN ’16^®^. Potential guest molecules were first docked within the cavity of the [Fe_2_**L**_3_]^4+^ complexes. The structures were energy minimized to give the optimized host-guest models. If the host-guest optimised models appeared to be capable of encapsulating multiple guest molecules, additional guests were docked within the cavity and the structures were once again optimised.


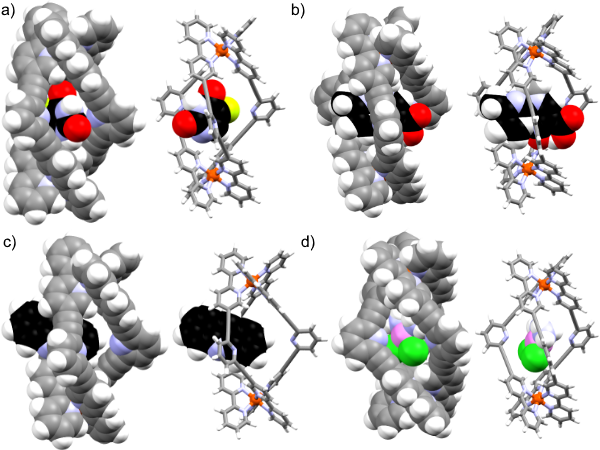


Figure S32 MMFF Spartan’16^®^ models of a) 5-fluorouracil ⊂ [Fe_2_(L1)_3_](BF_4_)_4_, b) nalidixic acid ⊂ [Fe_2_(L1)_3_](BF_4_)_4_, c) acridine ⊂ [Fe_2_(L1)_3_](BF_4_)_4_ and d) cisplatin ⊂ [Fe_2_(L1)_3_](BF_4_)_4_.


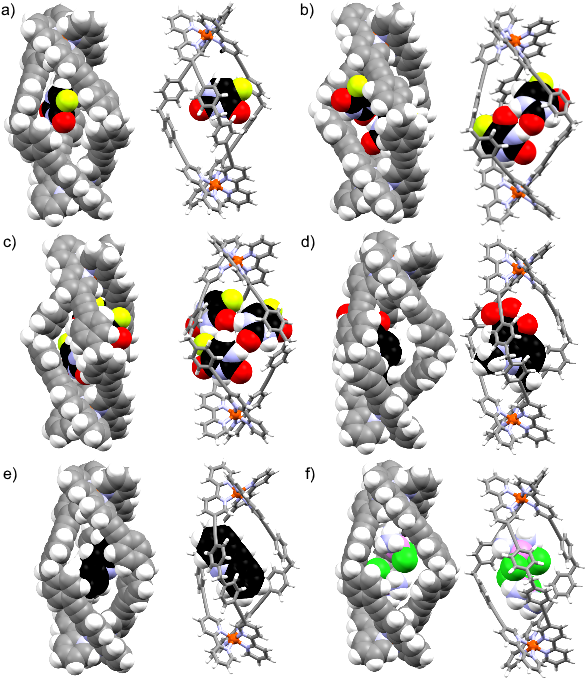


Figure S33 MMFF Spartan’16^®^ models of a) 5-fluorouracil ⊂ [Fe_2_(L2)_3_](BF_4_)_4_, b) (5-fluorouracil)_2_ ⊂ [Fe_2_(L2)_3_](BF_4_), c) (5-fluorouracil)_3_ ⊂ [Fe_2_(L2)_3_](BF_4_), d) nalidixic acid ⊂ [Fe_2_(L2)_3_](BF_4_)_4_, e) acridine ⊂ [Fe_2_(L2)_3_](BF_4_)_4_, f) cisplatin ⊂ [Fe_2_(L2)_3_](BF_4_)_4_

# Sulforhodamine B Cytotoxicity Assay.

HCT116, SW480 and NCI-H460 cells were supplied by ATCC, and SiHa cells were supplied by Dr. David Cowan, Ontario Cancer Institute, Canada. The cells were grown in α-MEM (Life Technologies) supplemented with 5% fetal calf serum (Moregate Biotech) at 37 °C in a humidified incubator with 5% CO_2_. The cells were seeded at 750 (HCT116, NCI-H460), 4000 (SiHa) and 5000 (SW480) cells/well in 96-well plates and left to settle for 24 h. Compounds were added to the plates in a series of 3-fold dilutions for 72 h before the assay was terminated by addition of 10% trichloroacetic acid (Merck Millipore) at 4 °C for 1 h. Cells were stained with 0.4% sulforhodamine B (Sigma-Aldrich) in 1% acetic acid for 30 min in the dark at room temperature and then washed with 1% acetic acid to remove unbound dye. The stain was dissolved in unbuffered Tris base (10 mM; Serva) for 30 min on a plate shaker in the dark and quantitated using a BioTek EL808 microplate reader at an absorbance of 490 nm with a reference wavelength of 450 nm to determine the percentage of cell-growth inhibition by determining the absorbance of each sample relative to a negative (no inhibitor) and a no-growth control (day 0). IC_50_ values were calculated with SigmaPlot 12.5 (Systat Software Inc.) using a three-parameter logistic sigmoidal dose−response curve between the calculated growth inhibition and the compound concentration. The presented IC_50_ values are the mean of at least 3 independent experiments, where 10 concentrations were tested in duplicate for each compound.

# X-ray Crystallography

CCDC #2064764, X-ray structure for [Fe_2_(**L1**)_3_](BF_4_)_4_. Vapor diffusion of diethyl ether into a concentrated solution of [Fe_2_(**L1**)_3_](BF_4_)_4_ in nitromethane yielded dark red needle shaped crystals. X-ray data was collected at 100 K on an Agilent Technologies Supernova system using Cu Kα radiation with exposures over 1.0^o^, and the data were treated using CrysAlisPro software. The structure was solved using SHELXT and weighted full matrix refinement on *F*^2^ was performed using SHELXL-97(Sheldrick, 2008) running within the OLEX2-v1.2.9 package.(Dolomanov et al., 2009) All non-hydrogen atoms were refined anisotropically, with hydrogen atoms attached to carbon atoms placed in calculated positions and refined using a riding model. The structure was solved in the space group P$\bar{1}$ and refined to an R_1_ value of 9.54%. The asymmetric unit contains two iron centres, three ligands (**L1**), three tetrafluoroborate anions and five nitromethane solvent molecules (Figure S28). A solvent mask (with the OLEX2‐v1.2.9 package) was applied to resolve diffuse electron density, where a void consisting of 373 electrons was measured, this was attributed to one tetrafluoroborate anion and ten nitromethane molecules.


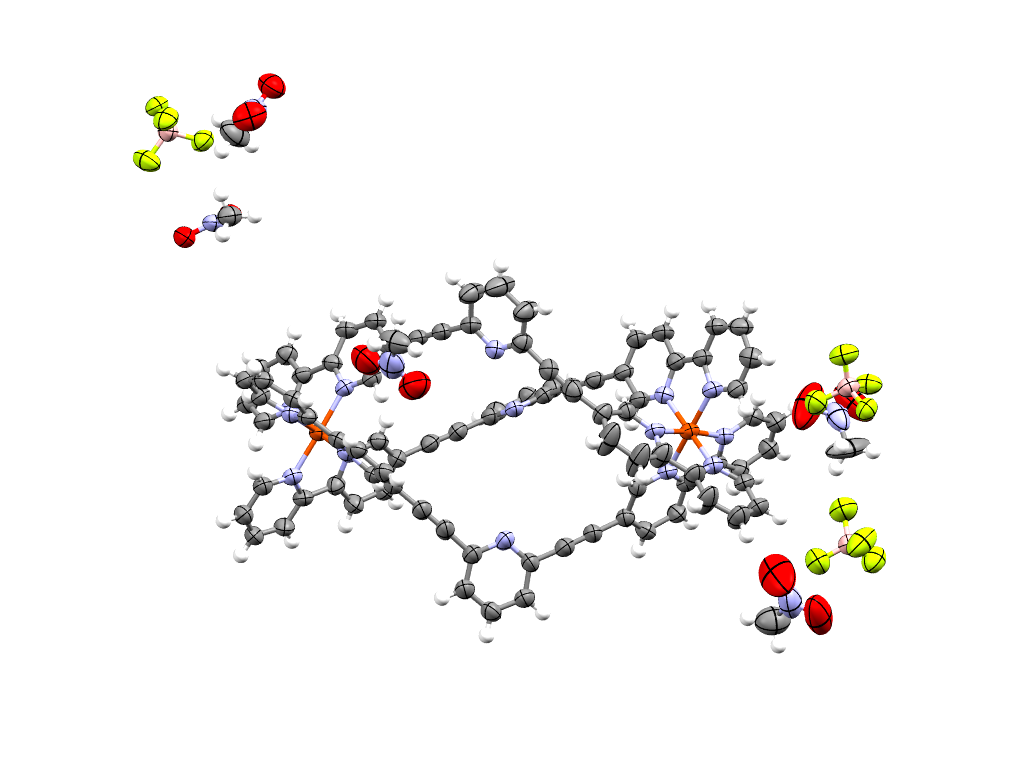


Figure S34 The mercury ellipsoid plot of the asymmetric unit of [Fe_2_(L1)_3_](BF_4_)_4_. Ellipsoids are shown at the 50% probability level. Colour scheme: carbon = grey, iron = red, fluorine = green, hydrogen = white, nitrogen = purple-blue, oxygen = red boron = salmon.

CCDC #2064761 X-ray structure for [Fe_2_(**L2**)_3_](BF_4_)_4_. Vapour diffusion of diethyl ether into a concentrated solution of [Fe_2_(**L2**)_3_](BF_4_)_4_ in nitromethane resulted in the formation of dark red cubic crystals. Data was collected at 100 K on an Agilent Technologies Supernova system using Cu Kα radiation with exposures over 1.0^o^, and the data were treated using CrysAlisPro software. The structure was solved using SHELXT and weighted full matrix refinement on *F*^2^ was performed using SHELXL-97(Sheldrick, 2008) running within the OLEX2-v1.2.9 package.(Dolomanov et al., 2009) All non-hydrogen atoms were refined anisotropically, with hydrogen atoms attached to carbon atoms placed in calculated positions and refined using a riding model. The structure was solved in the space group *P*2_1_/*n* and refined to an R_1_ value of 9.77%. The asymmetric unit contains two iron ions, three ligands (**L2**), two tetrafluoroborate anions and seven nitromethane solvent molecules (Figure S29). A solvent mask was applied to resolve diffuse electron density, where a large void consisting of 1154 electrons was measured, this was attributed to two tetrafluoroborate anions and thirty-three nitromethane solvent molecules.


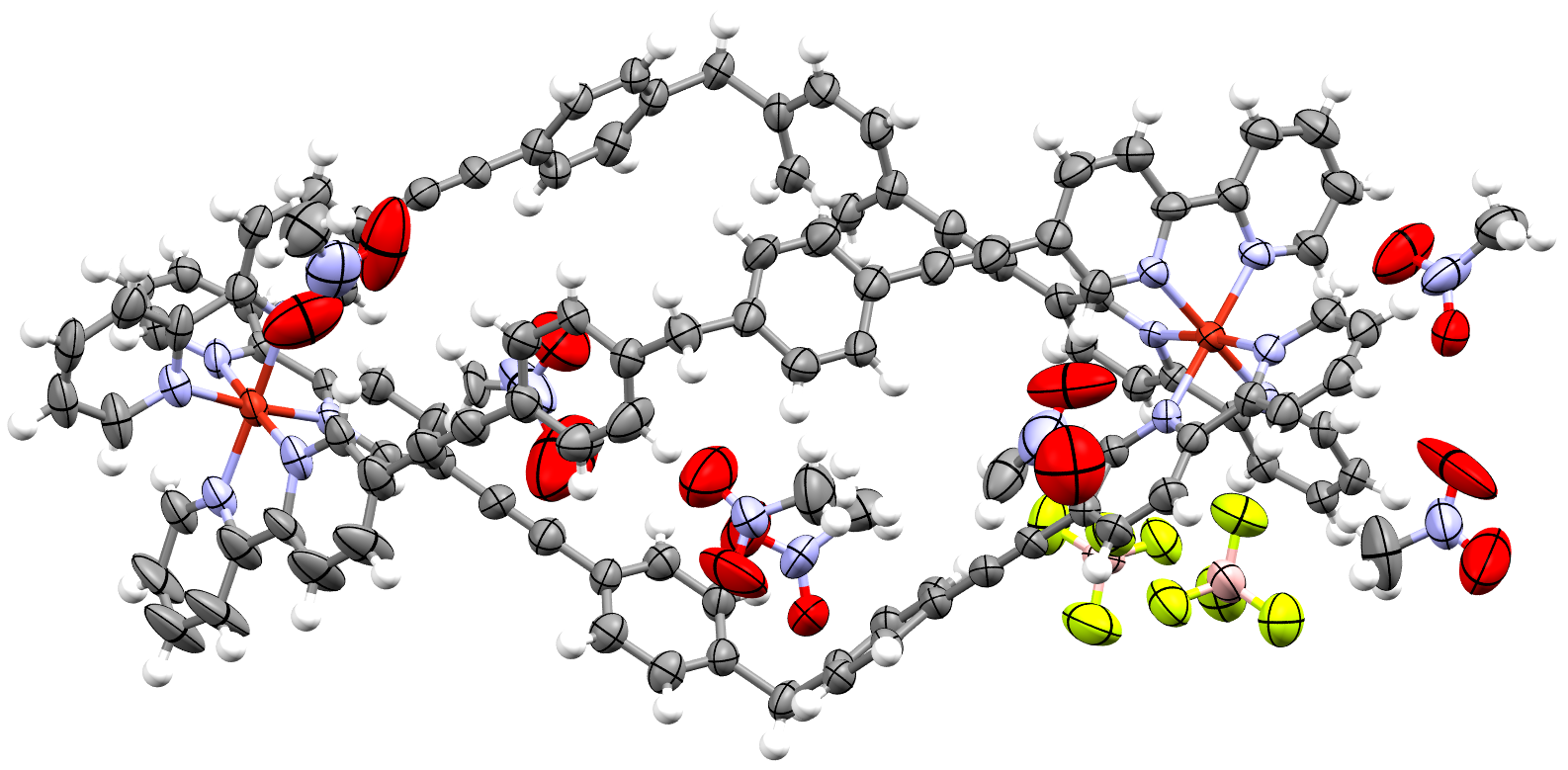


Figure S35 The mercury ellipsoid plot of the asymmetric unit of [Fe_2_(L2)_3_](BF_4_)_4_. Ellipsoids are shown at the 50% probability level. Colour scheme: carbon = grey, iron = red, fluorine = green, hydrogen = white, nitrogen = purple-blue, oxygen = red, boron = salmon.

## Table 2 X-ray data tables

| **Compound** | **Fe-N bond distances (Å)** | **Average Fe-N bond length (Å)** | **Fe-Fe distance(Å)** |
| --- | --- | --- | --- |
| [Fe_2_(**L1**)_3_](BF_4_)_4_ | **Fe1** 1.965, 1.951, 1.946, 1.959, 1.960, 1.978 | 1.960 | 14.16 |
|  | **Fe2** 1.948, 1.960, 1.988, 1.967, 1.962, 1.961 | 1.964 |  |
| [Fe_2_(**L2**)_3_](BF_4_)_4_ | **Fe1** 1.954, 1.964, 1.962, 1.953, 1.951, 1.949 | 1.956 | 19.12 |
|  | **Fe2** 1.988, 1.958, 1.966, 1.949, 1.965, 1.952 | 1.963 |  |
| [Fe_2_(**L_pyim_**)_3_](PF_6_)_4_  CCDC 622770 | **Fe1** 1.991, 1.972, 1.979, 1.980, 1.970, 1.986  **Fe2** 1.986, 1.980, 1.970, 1.979, 1.991, 1.972 | 1.973  1.980 | 11.41 |
|  |  |  |  |
| Fe(2,2'-bpy)](OTf)_2_ CCDC 1473638(Chàvez et al., 2016) | 1.960, 1.965, 1.973, 1.965, 1.959, 1.960 | 1.964 | - |
|  |  |  |  |

## Table 3 X-ray data tables

| **Identification code** | **[Fe_2_(L1)_3_](BF_4_)_4_** | **[Fe_2_(L2)_3_](BF_4_)_4_** |
| --- | --- | --- |
| **CCDC#** | 2064764 | 2064761 |
| **Empirical formula** | C_92_H_66_B_3_F_12_Fe_2_N_20_O_10_ | C_118_H_91_B_2_F_8_Fe_2_N_19_O_14_ |
| **Formula weight** | 1983.78 | 2284.41 |
| **Temperature/K** | 99.98(10) | 100.01(10) |
| **Crystal system** | triclinic | monoclinic |
| **Space group** | P$\bar{1}$ | *P*2_1_/*n* |
| **a/Å** | 17.0227(12) | 27.7535(4) |
| **b/Å** | 17.0919(11) | 18.1317(4) |
| **c/Å** | 19.2636 (13) | 28.1693(4) |
| **α/°** | 70.359(6) | 90 |
| **β/°** | 85.354(6) | 93.0190(10) |
| **γ/°** | 80.561(6) | 90 |
| **Volume/Å^3^** | 5205.2 (6) | 14155.6(4) |
| **Z** | 2 | 4 |
| **ρ_calc_ g/cm^3^** | 1.266 | 1.072 |
| **μ/mm^‑1^** | 2.955 | 2.212 |
| **F(000)** | 2026.0 | 4712.0 |
| **Crystal size/mm^3^** | 0.669 × 0.138 × 0.079 | 0.397 × 0.217 × 0.202 |
| **Radiation** | CuKα (λ = 1.54184) | CuKα (λ = 1.54184) |
| **2Θ range for data collection/°** | 7.072 to 129.472 | 7.956 to 123.782 |
| **Index ranges** | -19 ≤ h ≤ 19, -19 ≤ k ≤ 20, -22 ≤ l ≤ 21 | -31 ≤ h ≤ 31, -20 ≤ k ≤ 20, -31 ≤ l ≤ 32 |
| **Reflections collected** | 65147 | 105064 |
| **Independent reflections** | 17322 [R_int_ = 0.1361, R_sigma_ = 0.1117] | 21888 [R_int_ = 0.0512, R_sigma_ = 0.0428] |
| **Data/restraints/parameters** | 17322/6/1257 | 21888/90/1473 |
| **Goodness-of-fit on** *F*^2^ | 0.994 | 1.040 |
| **Final R indexes [I>=2σ (I)]** | R_1_ = 0.0954, wR_2_ = 0.2479 | R1 = 0.0977, wR2 = 0.2936 |
| **Final R indexes [all data]** | R_1_ = 0.1551, wR_2_ = 0.3020 | R_1_ = 0.1352, wR2 = 0.3303 |
| **Largest diff. peak/hole / e Å^-3^** | 0.72/-0.49 | 0.89/-0.52 |

# References

"CrysAlisPRO". (Yarnton, England: Oxford Diffraction /Agilent Technologies UK Ltd).

Austin, W.B., Bilow, N., Kelleghan, W.J., and Lau, K.S.Y. (1981). Facile synthesis of ethynylated

benzoic acid derivatives and aromatic compounds via ethynyltrimethylsilane. *J. Org. Chem.* 46**,** 2280-

2286.

Chàvez, J.E., Crotti, C., Zangrando, E., and Farnetti, E. (2016). Iron complexes with nitrogen bidentate

ligands as green catalysts for alcohol oxidation. *J. Mol. Catal. A: Chem.* 421**,** 189-195.

Dolomanov, O.V., Bourhis, L.J., Gildea, R.J., Howard, J.a.K., and Puschmann, H. (2009). OLEX2: a

complete structure solution, refinement and analysis program. *J. Appl. Crystallogr.* 42**,** 339-341.

Grosshenny, V., Romero, F.M., and Ziessel, R. (1997). Construction of Preorganized Polytopic Ligands

via Palladium-Promoted Cross-Coupling Reactions. *J. Org. Chem.* 62**,** 1491-1500.

Kim, S.-H., and Rieke, R.D. (2010). 2-Pyridyl and 3-pyridylzinc bromides: direct preparation and

coupling reaction. *Tetrahedron* 66**,** 3135-3146.

Scottwell, S.Ø., Elliott, A.B.S., Shaffer, K.J., Nafady, A., Mcadam, C.J., Gordon, K.C., and Crowley,

J.D. (2015). Chemically and electrochemically induced expansion and contraction of a ferrocene rotor.

*Chem. Commun.* 51**,** 8161-8164.

Sheldrick, G. (2008). A short history of SHELX. *Acta Crystallogr., Sect. A: Found. Crystallogr.* 64**,**

112-122.
